# Genomic variation between PRSV resistant transgenic SunUp and its progenitor cultivar Sunset

**DOI:** 10.1186/s12864-020-06804-7

**Published:** 2020-06-12

**Authors:** Jingping Fang, Andrew Michael Wood, Youqiang Chen, Jingjing Yue, Ray Ming

**Affiliations:** 1 The Public Service Platform for Industrialization Development Technology of Marine Biological Medicine and Product of State Oceanic Administration, Key Laboratory of Developmental and Neural Biology, College of Life Sciences, Fujian Normal University, Fuzhou, 350117 Fujian China; 2 Center of Engineering Technology Research for Microalgae Germplasm Improvement of Fujian, Southern Institute of Oceanography, Fujian Normal University, Fuzhou, 350117 Fujian China; 3 FAFU and UIUC-SIB Joint Center for Genomics and Biotechnology, Fujian Agriculture and Forestry University, Fuzhou, 350002 Fujian China; 4 Department of Plant Biology, University of Illinois at Urbana-Champaign, Urbana, IL 61801 USA

**Keywords:** *Carica papaya* L., Whole-genome resequencing, Genomic variation, Nuclear plastid DNA (NUPT), Nuclear mitochondria DNA (NUMT)

## Abstract

**Background:**

The safety of genetically transformed plants remains a subject of scrutiny. Genomic variants in PRSV resistant transgenic papaya will provide evidence to rationally address such concerns.

**Results:**

In this study, a total of more than 74 million Illumina reads for progenitor ‘Sunset’ were mapped onto transgenic papaya ‘SunUp’ reference genome. 310,364 single nucleotide polymorphisms (SNPs) and 34,071 small Inserts/deletions (InDels) were detected between ‘Sunset’ and ‘SunUp’. Those variations have an uneven distribution across nine chromosomes in papaya. Only 0.27% of mutations were predicted to be high-impact mutations. ATP-related categories were highly enriched among these high-impact genes. The SNP mutation rate was about 8.4 × 10^− 4^ per site, comparable with the rate induced by spontaneous mutation over numerous generations. The transition-to-transversion ratio was 1.439 and the predominant mutations were C/G to T/A transitions. A total of 3430 nuclear plastid DNA (NUPT) and 2764 nuclear mitochondrial DNA (NUMT) junction sites have been found in ‘SunUp’, which is proportionally higher than the predicted total NUPT and NUMT junction sites in ‘Sunset’ (3346 and 2745, respectively). Among all nuclear organelle DNA (norgDNA) junction sites, 96% of junction sites were shared by ‘SunUp’ and ‘Sunset’. The average identity between ‘SunUp’ specific norgDNA and corresponding organelle genomes was higher than that of norgDNA shared by ‘SunUp’ and ‘Sunset’. Six ‘SunUp’ organelle-like borders of transgenic insertions were nearly identical to corresponding sequences in organelle genomes (98.18 ~ 100%). None of the paired-end spans of mapped ‘Sunset’ reads were elongated by any ‘SunUp’ transformation plasmid derived inserts. Significant amounts of DNA were transferred from organelles to the nuclear genome during bombardment, including the six flanking sequences of the three transgenic insertions.

**Conclusions:**

Comparative whole-genome analyses between ‘SunUp’ and ‘Sunset’ provide a reliable estimate of genome-wide variations and evidence of organelle-to-nucleus transfer of DNA associated with biolistic transformation.

## Background

Papaya (*Carica papaya* L.) is a diploid plant with a relatively small genome (2*n* = 18, 372 Mb) in the family *Caricaceae* [1]. It is one of the most popular tropical fruits owing to its exceptional nutritional and medicinal properties. However, *Papaya Ringspot Virus* (PRSV) has been recognized as the most destructive disease threatening worldwide papaya production. In 1992, the papaya industry in Hawaii was devastatingly damaged and its marketable papaya production drastically declined as a result of the outbreak of PRSV [2]. The development of PRSV-resistant transgenic papaya ‘SunUp’ and ‘Rainbow’ revived the industry.

‘SunUp’ papaya is a genetically modified (GM) version of its non-GM progenitor ‘Sunset’, and the hybrid cultivar ‘Rainbow’ derived from crosses between ‘SunUp’ and ‘Kapoho’ became the first transgenic virus-resistant fruit tree cultivar to be commercialized in the United States [3]. Over 25 generations of inbreeding led to an extremely low genetic heterozygosity level of 0.06% in the red-fleshed cultivar ‘Sunset’ before transformation [4]. PRSV-resistant cultivar ‘SunUp’ was developed based on the concept of pathogen-derived resistance (PDR) through biolistic transformation of a plasmid vector containing the PRSV HA 5–1 coat protein (*cp*) gene expression cassette [5, 6]. ‘SunUp’ was obtained by selecting transgenic progenies that were homozygous for the *cp* functional transgene, which confer PRSV resistance [7]. ‘SunUp’ has grown apart from ‘Sunset’ for more than 25 generations, that is, more than 25 rounds of meiosis. A few differences are observed in modern ‘Sunset’ and ‘SunUp’ cultivars, although they share a lot of genetic features in common. In addition to the effects induced by transgene copy numbers and integration sites, other factors such as somaclonal variations during tissue culture and spontaneous mutations during meiosis of over 25 generations might induce segregated genomic variants, which would lead to the divergence of phenotypic and functional features between ‘Sunset’ and ‘SunUp’.

Genomic variants comprise small changes in nucleotides including single nucleotide polymorphisms (SNPs) and small insertion/deletions (InDels), and large changes in chromosome structure (> 50 bp), i.e. structural variants (SVs). SVs are considered to have a direct effect on behavior of the chromosome and cause variation in gene dosage [8]. Detection of genomic variants including unintended vector-derived fragments and other foreign fragments at the whole-genome level is characterized as an important criterion in the context of evaluation of GM organisms. The vector-derived inserts and transgene numbers in ‘SunUp’ were preliminarily determined by Southern analysis in a previous research [7], which revealed that three plasmid vector elements inserted in the host nuclear genome during bombardment were stably inherited afterwards. One was a 9789 bp functional insert, coding for intact functional transgenes PRSV *cp*, *nptII* and *uidA*; two were unintended and nonfunctional inserts, including a 290 bp partial *nptII* gene segment and a 1533 bp plasmid-derived fragment consisting of a 222 bp truncated *tetA* gene, respectively. Nevertheless, at the genome-wide structural level, it remains unclear what unintended alterations were induced during bombardment and tissue culture and how many spontaneous mutations accumulated in more than two decades of independent cultivation. Conventional Southern blot, PCR and comparative genome hybridization (array-CGH) techniques are the most prevalent methods applied in detection of exogenous DNA integration (> 20 bp), whereas other small unintended incorporations of exogenous DNA fragments are below the detection limit of these techniques.

In many eukaryotes, the host nuclear genomes are prevalently faced with the modification of themselves by integrations of their symbiotic organellar genomes [9–13]. Such transfers occur from both plastid and mitochondrial genomes to the nucleus and are termed nuclear plastid sequences (NUPTs) and nuclear mitochondrial sequences (NUMTs), respectively. The organelle-derived fragments in the nucleus are collectively known as nuclear organelle DNA (norgDNA). The gene content and genome complexity of nuclear genomes differs among angiosperm taxa typically associated with these continuing intercompartmental DNA transfer events [12]. In contrast to those beneficial or nonfunctional long-existing nuclear organelle integrations, substantial numbers of newly formed norgDNA are more deleterious and are rapidly eliminated [14, 15]. The pattern and mechanism of organelle-to-nucleus DNA transfer has been analyzed in detail in a number of species [16, 17]. NUPTs normally form continuous, inter/intra-chromosomal rearranged and mosaic structured patterns in the nuclear genome [18]. Non-homologous end joining of double-strand break repair (NHEJ-DSB repair) are suggested to be the integration mechanism as any other foreign sequences [18]. Recent evidence reveals that DNA methylation plays a pivotal role in regulating norgDNA, which may contribute to maintaining the genome stability and evolutionary dynamics of organellar and nuclear genomes [19]. NUPTs were shown to have integration preferences, simultaneous integration [20] and strong bias for nucleotide substitutions from C/G to T/A correlating with the time of integration [19]. It is intriguing that in Suzuki’s study [7] all six flanking genomic DNA segments of three transgenic inserts in ‘SunUp’ were nuclear organelle sequences. Five out of six were NUPTs, and one was NUMT. At present, no investigations have been conducted to determine whether bombardment affects the transfer frequency from cytoplasmic-to-nuclear genome or whether it was a consequence of insertion preference.

The last decade has witnessed revolutionary breakthroughs in next-generation sequencing (NGS) techniques, which enables fast and accurate re-sequencing of complete genomes at rather low costs. Whole-genome resequencing is a promising method for delivering information not only regarding inserts and their flanking sequences, but also about additional genome-wide assessments between genomes of transgenic lines versus their progenitors. The integration of norgDNAs and subsequent nucleotide changes can be detected by conducting sequence similarity analysis between nuclear organelle sequences and the organelle genomes, likewise their changes in distribution according to the time of integration can be easily estimated. The available papaya nuclear and organelle genome offer a distinct opportunity to study the genome-wide SVs and organelle-to-nucleus DNA shifts between GM papaya and its non-GM progenitor.

In the current study, we describe genome-wide comparative analysis of transgenic papaya ‘SunUp’ versus its progenitor ‘Sunset’, focusing on analysis of genomic variations such as small SNPs/InDels and large SVs, and the turnover and shuffling of nuclear organelle-derived sequences between the two varieties. These results will enable us to visualize the dynamic changes in ‘SunUp’ genome architecture after the integration of foreign sequences, provide evidence on where these norgDNA-like flanking sequences came from, and unravel the global impact of particle bombardment-mediated transformation on whole genome structure and organelle-to-nucleus DNA transfer.

## Results

### Whole-genome resequencing of ‘sunset’

The ‘Sunset’ genome was sequenced and assembled using a reference guided assembly approach using Illumina sequencing technology. The sequencing quality of these raw reads was generally high (90% with Phred quality score > 27). After filtering, a total of 74 million high quality, 124 bp paired-end (PE) reads were generated. The total read length was 9.197 Gb, representing around 24.72× genome equivalents (Table 1). The sequencing depths were evenly dispersed along the papaya chromosomes. We first mapped the PE reads back to the ‘SunUp’ reference genome by BWA’s short read aligner [21]. After removing multiple mapping reads and PCR duplicates, 48 million clean reads were retained for the following study. Of these ‘Sunset’ reads, as high as 99.97% matched unique ‘SunUp’ genomic locations, showing substantial consistency over most genome regions between ‘SunUp’ and ‘Sunset’. The remaining 15,822 reads (0.03%) were unmapped, and likely correspond to the organelle genomes, ‘Sunset’-specific region or highly repetitive regions that were unassembled in the reference ‘SunUp’ genome. Approximately 46 million (95.78%) clean reads mapped to reference genome in a properly paired orientation.

**Table 1:** Papaya Sunset genome-wide sequencing and mapping statistics

		Sunset genome wide
	Total read count	74,169,662
	Read length (bp)	124
	Total read length (Gb)	9.197
	Average coverage (×)	24.72
Remove multiple mapping and duplicates	Total read count	48,170,821
	Mapped read count	48,154,999
	Mapped read rate (%)	99.97
	Unmapped read count	15,822
	Properly paired read count	46,139,627
	Properly paired read rate (%)	95.78

### Detection and characterization of SNPs, small InDels and large SVs in ‘sunset’

Polymorphisms between ‘Sunset’ and ‘SunUp’ were identified using SAMtools software suite [22] with strict parameters. Polymorphisms with coverage < 10 or > 100 and quality < 50 were discarded to eliminate false positives in low coverage and highly repetitive regions respectively. Polymorphism sites with only one ALT were retained given the diploid nature of papaya. In total, 310,364 SNPs and 34,071 small InDels were found between ‘Sunset’ and the ‘SunUp’ reference genome (Table 2), with an average mutation rate of 0.084% for SNPs vs. 0.009% for InDels. The number of heterozygous SNPs was nearly 7 times higher than that of homozygous SNPs (269,493 vs. 40,871). A more even distribution was observed in the numbers of homozygous and heterozygous InDels, with 19,135 and 14,936, respectively. The genome wide average for polymorphisms across the ‘Sunset’ genome was 84 SNPs per 100 kb and 9 InDels per 100 kb (Table 3 and Fig. S1). SNPs were substantially more prevalent at the genome-wide level than InDels. SNPs had an uneven distribution across the nine chromosomes of papaya ranging from 24 SNPs per 100 kb in chromosome 2 to 165 SNPs per 100 kb in chromosome 6. InDels were more evenly dispersed across the ‘Sunset’ genome ranging from an average of 7 InDels per 100 kb in chromosome 2/9 to 13 InDels per 100 kb in chromosome 6.

**Table 2:** Number of homo/hetero SNPs and InDels detected before and after data filtering

	Raw	DP10-100Q50^**a**^
Homo SNPs	83,926	40,871
Hetero SNPs	603,970	269,493
**Total SNPs**	687,896	**310,364**
Homo InDels	41,218	19,135
Hetero InDels	29,504	14,936
**Total InDels**	70,722	**34,071**
**Total**	758,618	**344,435**

Notes: (^a^): Validated depth and quality. DP10-100Q50: The variant calls with read depths of < 10 or > 100 and polymorphism sites of quality < 50 were filtered out

**Table 3:** Summary of polymorphisms between SunUp and Sunset

Chrom.	Total size(bp)	No.of SNPs	No.of InDels	SNP per 1 kb	In/Del per 1 kb
CHROM_1	22,976,894	16,246	2214	0.71	0.10
CHROM_2	28,675,255	6842	1893	0.24	0.07
CHROM_3	29,397,938	18,294	2630	0.62	0.09
CHROM_4	27,056,416	12,813	2426	0.47	0.09
CHROM_5	24,352,217	13,952	2150	0.57	0.09
CHROM_6	30,516,430	50,463	3821	1.65	0.13
CHROM_7	22,375,162	17,294	2361	0.77	0.11
CHROM_8	21,952,264	12,610	2001	0.57	0.09
CHROM_9	27,303,179	12,021	1986	0.44	0.07
Unanchored scaffolds	135,176,073	149,829	12,589	1.11	0.09
Genome-wide	369,781,828	**310,364**	**34,071**	0.84	0.09

All types of base changes were obtained and subdivided into transitions (Ts) and transversions (Tv) (Table 4, Fig. S2). The total amount of Ts and Tv detected in all SNPs was 205,333 and 105,031 respectively. The Ts/Tv ratio was 1.95. The average ratios of Ts to Tv for homozygous and heterozygous SNPs were 1.03 and 2.18, respectively. The amount of all four types of Ts were observed to have between 3.4- to 5.8-fold more than that of any types of Tv. The SNPs consisted of 104,312 G/C to A/T transitions (33.6%), 101,021 A/T to G/C transitions (32.6%), followed by 29,222 G/C to T/A transversions (9.4%), 28,910 A/T to C/G transversions (9.3%), 28,835 A/T to T/A (9.3%) and 18,064 G/C to C/G transversions (5.8%). Changes from G/C to A/T (Ts) were observed with the highest frequency whereas G/C to C/G (Tv) were the least frequent changes.

**Table 4:** Pattern of homozygous and heterozygous SNPs

SNP pattern		Homo SNPs	Hetero SNPs	Total SNPs
Transition	A/G	5315	45,067	50,382
	T/C	5768	44,871	50,639
	G/A	4701	47,543	52,244
	C/T	4908	47,160	52,068
	**total(Ts)**	**20,692**	**184,641**	**205,333**
Transversion	A/C	2329	12,114	14,443
	A/T	2327	11,999	14,326
	T/A	2310	12,199	14,509
	T/G	2274	12,193	14,467
	G/C	2509	6589	9098
	G/T	3020	11,576	14,596
	C/A	3104	11,522	14,626
	C/G	2306	6660	8966
	**total(Tv)**	**20,179**	**84,852**	**105,031**
	**Ts/Tv**	**1.03**	**2.18**	**1.95**

**Fig. 1:**
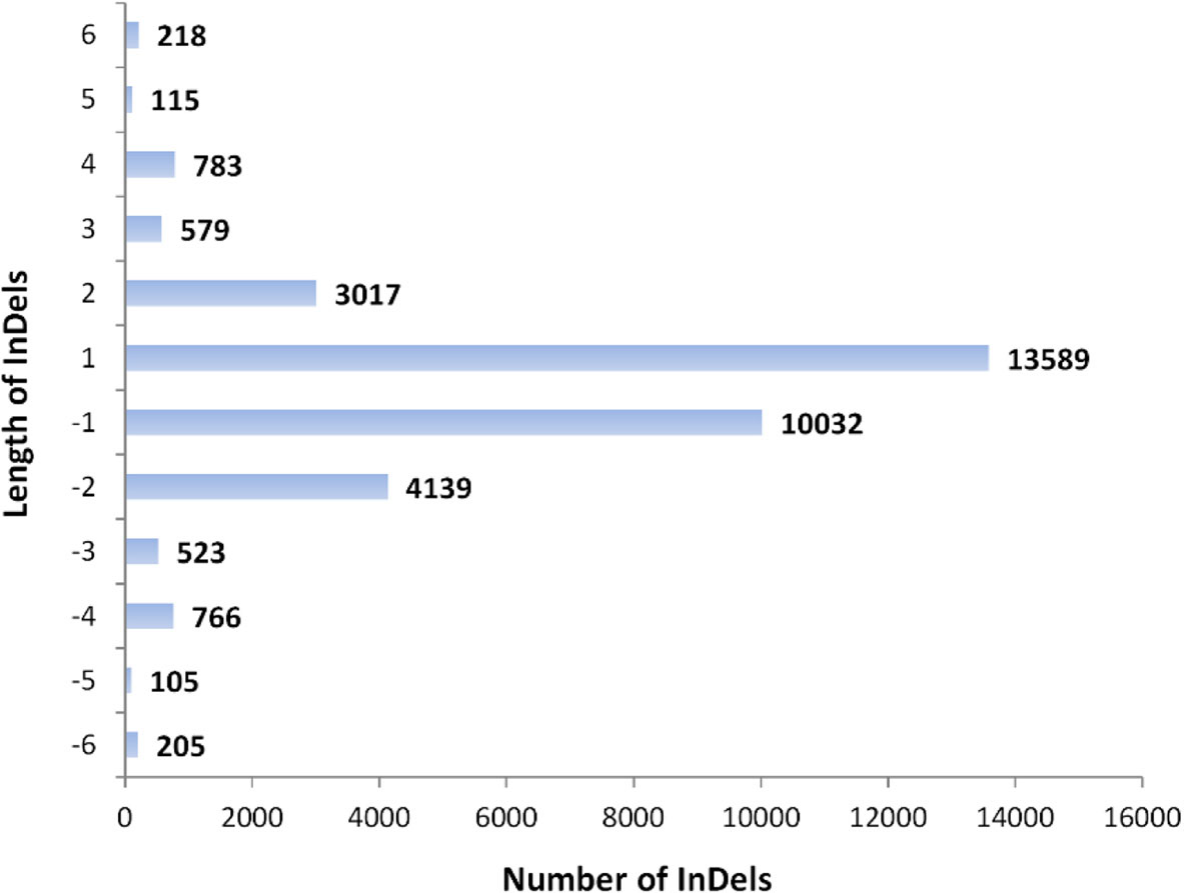
Histogram of InDels number and length in Sunset genome compared to SunUp reference genome

The length of small InDels ranged in size from 1 to 6 bp throughout the entire genome (Fig. 1), of which 1 bp-sized InDels were the most abundant, followed by 2 bp-sized InDels. In general, the amount of InDels decreased sharply as their size increased, especially for the shortest ones (1- to 2-bp) which showed the most dramatic drop in number. An exception was that the number of 3 bp-sized and 5 bp-sized InDels were slightly less than that of 4 bp-sized and 6 bp-sized InDels respectively.

The BLAST result indicated that no additional plasmid derived inserts were found in the available ‘SunUp’ genome with the exception of three previously detected plasmid-derived inserts. In addition to SNPs and small InDels, the prevalence of some other types of larger structural variations (> 50 bp) such as larger insertions (INS) and deletions (DEL), inversions (INV), intra-chromosomal translocations (ITX) and inter-chromosomal translocations (CTX) were also assessed using BreakDancer under stringent criteria. A total of 1200 structural variants were identified in ‘Sunset’ (Table S1). These SVs were further validated by manual inspection of ‘Sunset’ paired-end read alignments. We observed that all of SVs were unreliably predicted or false positives. Although each detected SV was supported by several reads, these regions were also covered by paired-end reads that matched the arrangement of papaya ‘SunUp’ reference genome. All false positives were found to be located in the gap regions or regions with high levels of coverage (> 100).

### Classification of SNPs and small InDels by potential impact on protein function

**Fig. 2:**
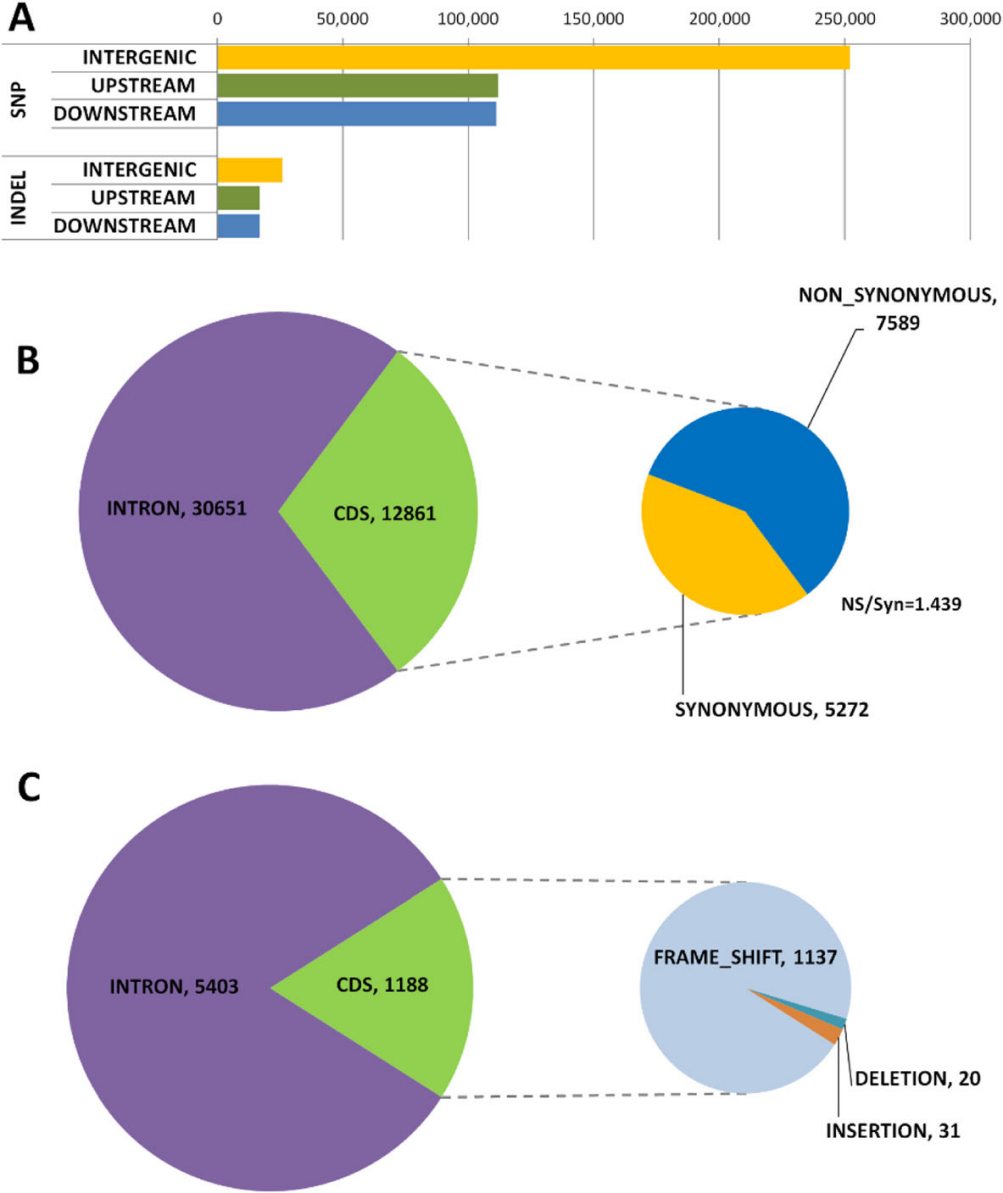
Annotation of single-nucleotide polymorphisms (SNPs) and InDels in Sunset genome compared to SunUp reference genome. **a**. Distribution of SNPs and InDels in intergenic, upstream and downstream regions. **b**. Distribution of SNPs in different genic regions. **c**. Distribution of InDels in genic regions. The number of synonymous and non-synonymous SNPs detected within the CDS region has also been shown

We predicted the variant effects of SNPs and small InDels according to their potential impact on protein function using SNPEff program [23] and self-built papaya data sets (Fig. 2 and Table 5). All variants that may have an effect on protein function could be categorized into 35 effect types, which were further grouped into the following four larger predefined impact categories on the basis of the assumed severity: HIGH, MODERATE, LOW, and MODIFIER (Table 5). The vast majority of variants (571,039, 97.4%) belonged to the MODIFIER category, which is usually comprised of intronic and intergenic variants and assumed to have only a weak or no impact on the protein. The LOW category is thought to be mostly harmless or unlikely to change protein behavior, such as synonymous mutations. A non-disruptive variant that might change protein effectiveness is defined as MODERATE, including in-frame deletions and missense mutations. In all 7533 (1.28%) and 6114 (1.04%) variants had possible MODERATE and LOW impacts on gene function. Only 1591 variants with HIGH impacts were found, representing 0.27% of the total variants, which are assumed to have disruptive impacts on the protein, probably causing protein truncations, loss of function or triggering nonsense mediated decay. The most common types of mutations were frameshift variants in the HIGH category.

**Table 5:** Prediction of the effects of SNPs and InDels

Impact (count, percentage in Sunset)	Effect type	Count	Percentage (%)
**HIGH (1591, 0.2714%)**	frameshift_variant	1033	0.1762
	frameshift_variant+splice_region_variant	66	0.0113
	frameshift_variant+start_lost	12	0.0020
	frameshift_variant+stop_gained	9	0.0015
	frameshift_variant+stop_gained+splice_region_variant	1	0.0002
	frameshift_variant+stop_lost	1	0.0002
	frameshift_variant+stop_lost+splice_region_variant	15	0.0026
	splice_acceptor_variant+intron_variant	75	0.0128
	splice_acceptor_variant+splice_region_variant+intron_variant	2	0.0003
	splice_donor_variant+intron_variant	87	0.0148
	splice_donor_variant+splice_region_variant+intron_variant	1	0.0002
	start_lost	24	0.0041
	start_lost+splice_region_variant	1	0.0002
	stop_gained	185	0.0316
	stop_gained+disruptive_inframe_insertion	1	0.0002
	stop_gained+splice_region_variant	6	0.0010
	stop_lost	23	0.0039
	stop_lost+inframe_insertion+splice_region_variant	1	0.0002
	stop_lost+splice_region_variant	48	0.0082
**MODERATE (7533, 1.2849%)**	missense_variant+splice_region_variant	130	0.0222
	disruptive_inframe_deletion	3	0.0005
	disruptive_inframe_insertion	7	0.0012
	inframe_deletion	17	0.0029
	inframe_insertion	22	0.0038
	missense_variant	7354	1.2544
**LOW (6114, 1.0429%)**	initiator_codon_variant	9	0.0015
	splice_region_variant+intron_variant	833	0.1421
	splice_region_variant+stop_retained_variant	13	0.0022
	splice_region_variant+synonymous_variant	100	0.0171
	stop_retained_variant	4	0.0007
	synonymous_variant	5155	0.8793
**MODIFIER (571,039, 97.4009%)**	downstream_gene_variant	128,197	21.8663
	intergenic_region	278,076	47.4308
	intron_variant	36,054	6.1497
	upstream_gene_variant	128,712	21.9541

Notes: Variants (SNPs and InDels) that may affect protein function were categorized into 35 types. These types were further grouped into HIGH, MODERATE, LOW, and MODIFIER according to potential severity. The assignment criteria were pre-defined in the annotation program (SNPEff)

In terms of genomic distribution, intergenic regions contained high proportions of SNPs, accounting for approximately 48.5% while merely 8.4% were identified in genic regions. About 21% were present in upstream promoter regions and downstream regulatory regions (Fig. 2a). Within the genic region, 2.5 and 5.9% of SNPs were present in the coding sequence (CDS) regions and introns, respectively (Fig. 2b). Overall, SNPs and InDels were spread over the entire genome with a similar distribution pattern. Likewise, a substantial number of InDels (~ 39%) were identified in intergenic regions (Fig. 2a), whereas only 9.9%were located in genic regions, consisting of 8.1% of intronic InDels and 1.8% of exonic InDels (Fig. 2a). The presence of InDels in the upstream and downstream regulatory regions of genes was also shown with a relatively high percentage (~ 25%) (Fig. 2a). In order to investigate the effect of SNPs on the amino acid alteration of a protein, the likelihood of non-synonymous and synonymous coding SNPs was estimated. Among all SNPs, 7589 non-synonymous and 5272 synonymous type modifications were detected in ‘Sunset’ (Fig. 2b). The ratio of non-synonymous to synonymous SNPs (NS/Syn ratio) was about 1.439. The predominant InDels within the coding regions were frameshift mutations (1137, 95.7%), i.e. an indel size of which is not multiple of 3 (the length of a codon), whereas a significantly lower amount of codon insertions (31, 2.6%) and deletions (20, 1.7%) was observed (Fig. 2c).

With respect to gene function, all high-impact SNPs were predicted to affect 1454 genes. For the global functional analysis of HIGH category genes, Gene Ontology (GO) terms were assigned to corresponding genes using BLAST2GO software [24]. Of 1454 high-impact genes, 751 genes were associated with at least one GO term. GO category enrichment analysis was further performed to elucidate the functional enrichment of potentially high-impact genes, using Fisher’s exact test with an FDR cutoff ≤0.05. There were 31 GO terms significantly enriched in biological processes and molecular functions (See Table S2 and Fig. S3). Those high-impact genes most significantly enriched in the biological process GO term “ATP catabolic process”, followed by “ribonucleotide catabolic process”, and “purine nucleotide catabolic process”. A number of related molecular function GO terms were significantly enriched, including “nucleoside-triphosphatase activity”, “hydrolase activity, acting on acid anhydrides, in phosphorus-containing anhydrides” and “ATPase activity”, etc.

### Shared and specific nuclear organelle integration sites between ‘SunUp’ and ‘sunset’

**Fig. 3:**
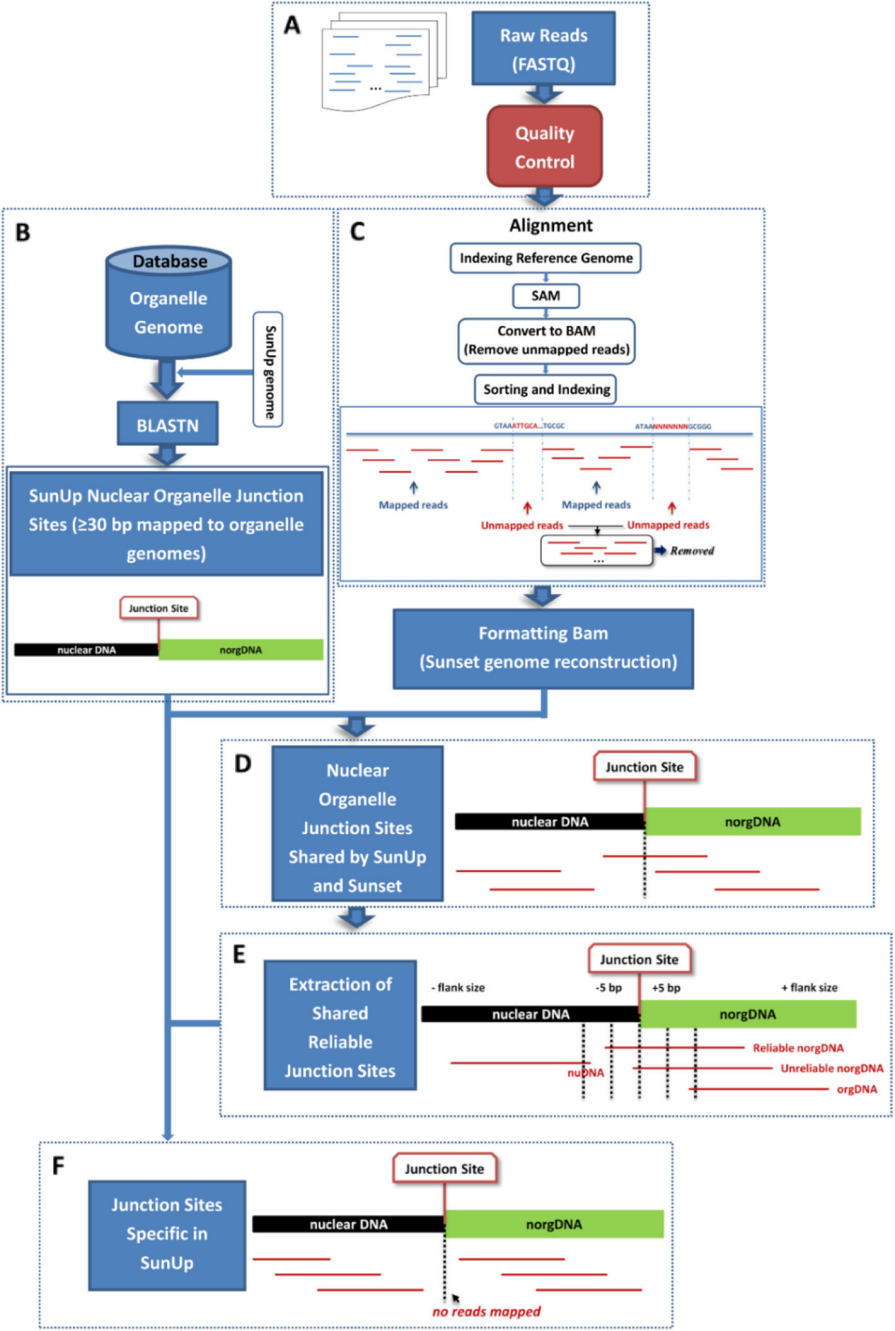
Pipeline of SunUp-specific genomic integration of nuclear organelle DNA fragments.** a**. Quality control of raw sequenced data. **b**. Searches for SunUp nuclear organelle junction sites by BLASTN [25]. The BLASTN algorithm was used to search SunUp genome for nuclear plastid DNA (NUPT) and nuclear mitochondria DNA (NUMT) integrations with papaya organelle genomes as databases. Only hits with ≥30 bp mapped to organelle genomes were considered. **c**. Alignment between Sunset reads and SunUp reference genome. Unmapped reads were removed after subsequent analysis. **d**. Nuclear organelle junction sites shared by SunUp and Sunset. A junction site was supposed to be shared by SunUp and Sunset genomes when there were reads mapped to and spanning its position in the SunUp reference genome. **e**. Extraction of reliable shared junction sites. The mixture of reads that aligned back to the reference genome may originate from different sources of DNA in the Sunset genome, including nuclear DNA (nuDNA), nuclear organelle DNA (norgDNA) and organelle DNA (orgDNA). In order to discriminate these three categories of reads and extract the reliable junction sites shared by SunUp and Sunset, the flanking regions (5 bp upstream and downstream) of the junction sites are used as an indicator. Reliable norgDNA reads were selected if those reads were spanning the junction sites and mapped to at least 5 bp of norgDNA or nuDNA. **f**. Junction sites specific in SunUp. If there were no reads mapped to or no reliable norgDNA reads spanning the junction site, we considered this junction site as a SunUp-specific norgDNA junction site

**Fig. 4:**
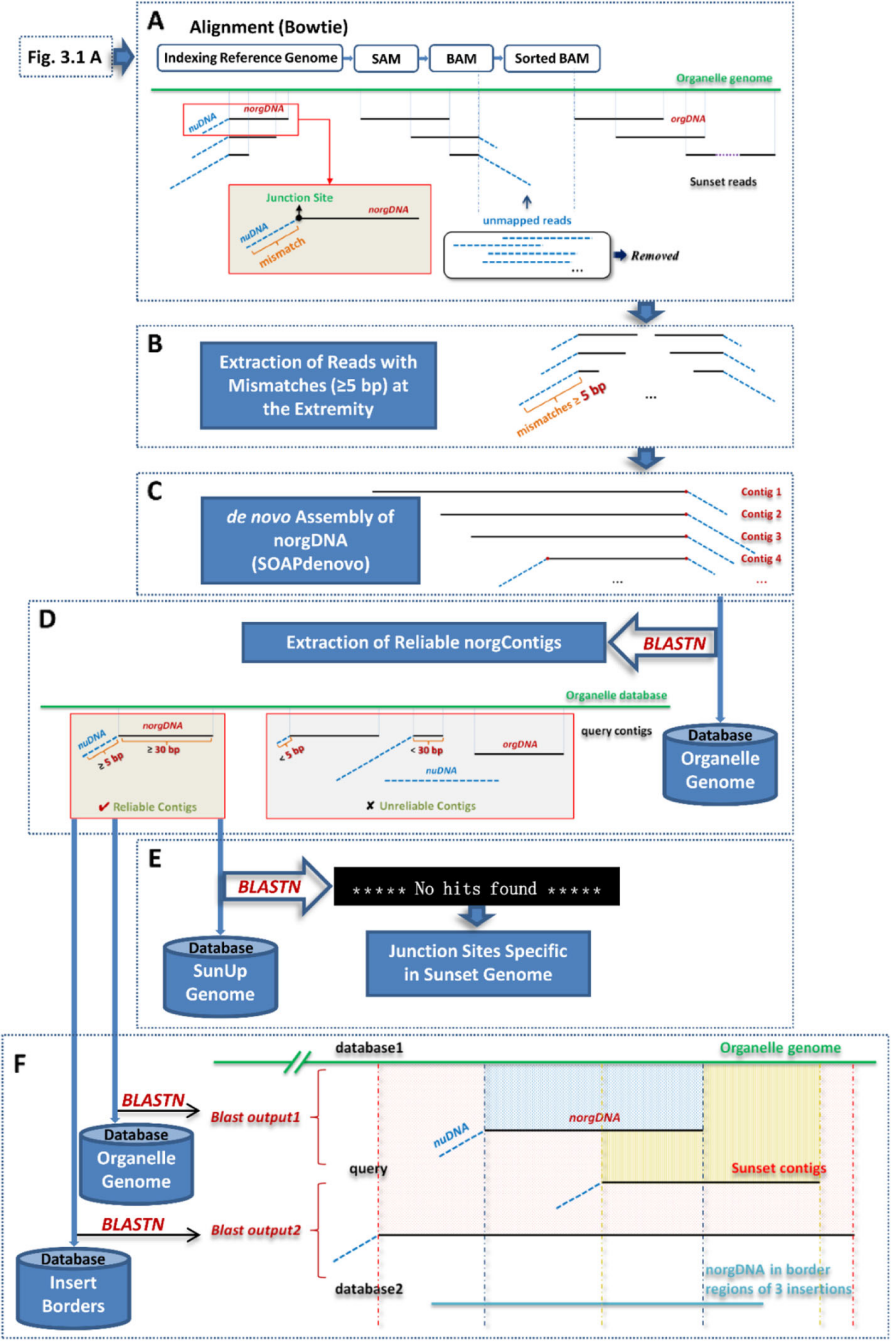
Pipeline of Sunset-specific genomic integration of nuclear organelle DNA fragments. **a**. Alignment between Sunset reads and organelle reference genome. Unmapped reads were removed after subsequent analysis. Soft-clipped reads were shown in the red box, which refers to reads with mismatches at the extremities. **b**. Extraction of reads with at least 5 bp mismatches (≥5 bp) at the extremities. **c**. de novo assembly of norgDNA by SOAPdenovo. **d**. Extraction of reliable Sunset norgContigs. Only blast hits of norg contigs with ≥30 bp mapped to organelle genomes and ≥ 5 bp unmatched on the edges were considered as reliable norgContigs. **e**. Junction sites specific in Sunset. The Sunset-specific norg sequences were obtained when no hits were determined using BLAST against the SunUp reference genome. **f**. Identity between the six organelle-like borders of transgenic insertions in SunUp and Sunset norgDNA

With the aim of conducting genome-wide comparative analysis of the integration of nuclear organelle fragments between ‘SunUp’ and ‘Sunset’, two in-house software pipelines written in a mixture of python scripts (available upon request) were developed for automatic processing and identification of shared and variety-specific norgDNA integration sites between these two varieties. Schematic diagrams of pipelines are shown in Fig. 3 and Fig. 4.

A total of 3430 NUPT and 2764 NUMT junction sites were obtained by searching against organelle genomes with the ‘SunUp’ reference genome as the query (Table 6). Out of all 3430 NUPT junction sites, a large fraction of junction sites (3327, 97%) were shared by ‘SunUp’ and ‘Sunset’. With BLASTN we identified that shared NUPTs matched the papaya chloroplast (pt) genome with an average identity of 91.92%. The remaining 3% (103) were specific in ‘SunUp’, with a higher average identity of 94.03% to the pt. genome (further details of the 103 junction sites are provided in Table S3). Similar to the trend observed for the distribution of NUPTs, out of 2764 NUMT junction sites, junction sites shared between ‘SunUp’ and ‘Sunset’ numbered 2642 and account for the major share 95.6% whereas ‘SunUp’-specific junction sites only accounted for 4.4% (122) (further details of the122 junction sites are provided in Table S4). The average similarity in identity between ‘SunUp’-specific NUMTs and papaya mitochondria (mt) genome was 93.77%, which is slightly less than the identity between ‘SunUp’-specific NUPTs and the pt. genome (94.03%) but a bit higher than the identity between shared NUMTs and the mt genome (92.97%). In general, higher similarities in identities were apparent between ‘SunUp’-specific norgDNAs and corresponding organelle genomes than between shared norgDNAs and corresponding organelle genomes. We next evaluated the performance of our pipeline through manual inspection of read alignments surrounding those identified as ‘SunUp’-specific norgDNA junction sites in the Integrative Genomics Viewer (IGV) software [26]. The visual display exhibited that no ‘Sunset’ reads aligned to or spanned any ‘SunUp’-specific junction site in the ‘SunUp’ reference genome as we had expected, thus those ‘SunUp’-specific integration events predicted by our pipeline were bona fide. In the ‘SunUp’-specific norgDNA regions, no reads mapped or having a read depth greater than 100× were observed, suggesting that those reads likely correspond to the organellar DNA. The results demonstrate the superior sensitivity and accuracy of our pipeline.

**Table 6:** Junction site numbers and identities of NUPT and NUMT

Junction site type	NUPT			NUMT		
	Count	Percentage	Identity (nupt/pt)^a^	Count	Percentage	Identity (numt/mt)^a^
SunUp	3430	100.00%		2764	100.00%	
Shared	3327	97.00%	91.92%	2642	95.59%	92.97%
Specific in SunUp	103	3.00%	94.03%	122	4.41%	93.77%
Sunset	3346	100.00%		2745	100.00%	
Shared	3327	99.43%	91.92%	2642	95.50%	92.97%
Specific in Sunset	19	0.57%	95.64%	103	4.50%	96.95%

Notes: (^a^): the identity between nupt/numt and corresponding organelle genome. Chloroplast (pt); mitochondria (mt)

Overall, ‘SunUp’-specific norgDNA integration junction sites were distributed non-randomly across nine chromosomes of papaya, with distinct regions of high and low variation (Table 7). The most distinct region was in Chr2 which had the highest frequency of NUPT junction sites with 11.65% compared to other chromosomes of the genome, followed by Chr6 and Chr8, with 8.74% each. Only a low proportion of NUPT junction sites were found in Chr3 (1.94%) and Chr2 (2.91%). Compared with NUPT junction sites, a smaller range of variation across chromosomes was found at NUMT junction sites. Similarly, NUMT junction sites were highly enriched in Chr6 (10.66%), Chr2 (9.84%) and Chr8 (9.02%), while less prevalent in Chr5 (4.92%) and Chr1 (5.74%).

**Table 7:** The chromosome information for organelle DNA integration sites

Chromosome	Specific junction sites in SunUp			
	NUPT		NUMT	
	**Count**	**Percentage**	**Count**	**Percentage**
CHROM_1	3	2.91%	7	5.74%
CHROM_2	12	11.65%	12	9.84%
CHROM_3	2	1.94%	8	6.56%
CHROM_4	9	8.74%	10	8.20%
CHROM_5	6	5.83%	6	4.92%
CHROM_6	9	8.74%	13	10.66%
CHROM_7	6	5.83%	10	8.20%
CHROM_8	9	8.74%	11	9.02%
CHROM_9	8	7.77%	8	6.56%
Unanchored scaffolds	39	48.75%	37	30.33%
Total	103	100.00%	122	100.00%

Using a strict pipeline (Fig. 4), the ‘Sunset’ genome was also scanned for norgDNA integrations by searching the papaya chloroplast and mitochondria genomes. The total amount of either NUPT or NUMT integration junction sites in the ‘Sunset’ genome were slightly fewer than in the ‘SunUp’ genome, with 3430 NUPT and 2764 NUMT junction sites, respectively (Table 6). In contrast to ‘SunUp’-specific NUPT integrations (103), the amount of ‘Sunset’-specific NUPT integration junction sites sharply reduced to only 19, with an average sequence identity of 95.64% matching to the papaya pt. genome; ‘Sunset’-specific NUMT integration junction sites decreased to 103, having an average identity of 96.95% to the mt genome.

### The origin of organelle-like borders of transgenic inserts in ‘SunUp’

**Fig. 5:**
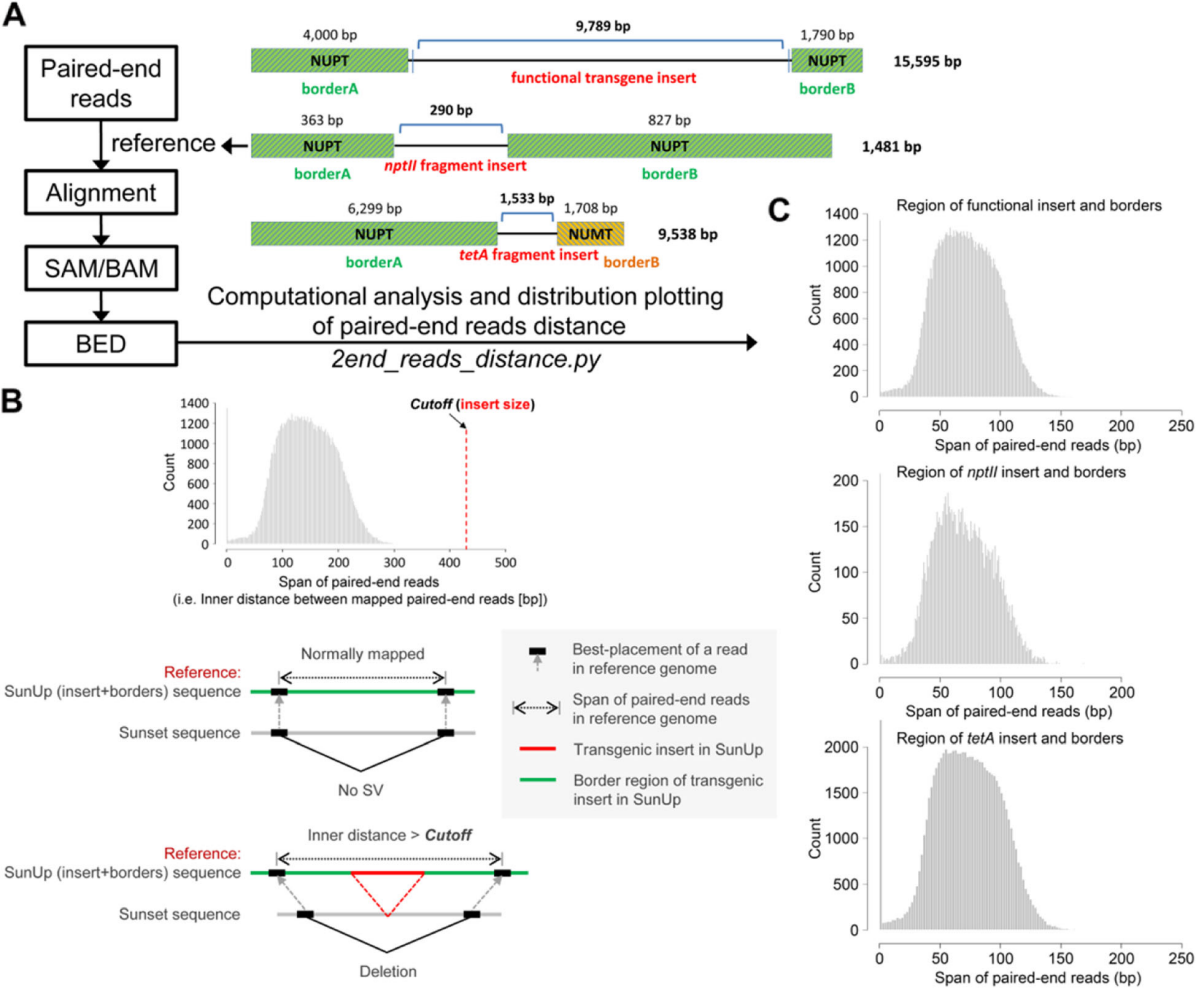
Workflow for the identification of the origin of the flanking norgDNA of transgenic inserts. **a**. Sequences of three SunUp transformation plasmid derived inserts with borders and the bwa alignment process. **b**. A strategy using high-throughput and massive paired-end mapping to identify deletions in Sunset relative to the reference genome. Insertions in SunUp were predicted from paired-end spans larger than a specified cutoff (size of a transgenic insert). **c**. Histogram plots exhibiting the inner distance of mapped paired-ends in regions of three inserts with borders

BLASTN search analysis of transgenic inserts’ flanking sequences was conducted to investigate the possible identity of sequences around the insertion sites. All six genomic DNA segments flanking the three previously identified transgenic insertions were surprisingly found to share near sequence identity to the papaya organelle sequences (Fig. 5a). Both sides of the single, contiguous 9789 bp functional transgene insertion encoding intact PRSV *cp*, *uidA* and *nptII* genes were identified to be NUPT sequences, consisting of a 4000 bp and a 1, 790 bp plasmid-derived segments, which were highly homologous with *trn*, *rps* genes of the plasmid genome and part of the *ycf3* gene. The genomic DNA flanking both borders of the nonfunctional *nptII* fragment insert (290 bp) also exhibited homology with papaya plastid genome genes *ndhG* and *atpB, E*, with size of 363 bp and 827 bp, respectively. The contiguous 1533 bp nonfunctional *tetA* fragment insert, in particular, had one border of NUPT sequence homologous to the plastid gene *ycf2*, reaching up to 6199 bp. The other border of the *tetA* fragment was comprised of non-plastid DNA-like sequence and showed identity to a papaya mitochondria genome segment, totaling 1708 bp. Sequences of three flanking pairs of transgenic inserts showed significant homology to papaya organelle genome segments, with a range of 98.18 ~ 100% identity. Especially two flanking pairs of the functional transgene insert and the nonfunctional *nptII* fragment insert, having identities approaching 100%. By contrast, organelle-like sequences at both borders of the *tetA* fragment insert experienced further rearrangements and showed lower similarities of 98.6 and 98.18% with the pt. genome and the mt genome, respectively. We estimated the homology between our previously assembled ‘Sunset’ norgDNAs and six flanking organelle-like sequences of inserts in ‘SunUp’. Through a rigid BLAST screening, there were respectively 12, 6, 1, 5, 43 and 2 best BLAST hits detected between ‘Sunset’ norgDNAs and six flanking norgDNAs, with combined lengths ranging from 49 to 4180 bp (Table 8). Only one (best) hit was found between ‘Sunset’ norgDNAs and border A of the nonfunctional *nptII* fragment insert, with a size of 49 bp; whereas there was at least 1231 bp of combined length for the remaining five borders. The sequence identity between ‘Sunset’ norgDNAs and six flanking norgDNAs of insertions in ‘SunUp’ varied from 93.68 to 99.09%. Of which all NUPT borders matched ‘Sunset’ norgDNAs with relatively lower identities in comparison to their matching with the papaya chloroplast. Meanwhile, the sole NUMT border had 99.09% similarity to ‘Sunset’ norgDNAs, which is higher than its similarity to the mt genome (98.18%).

**Table 8:** Comparative analysis of 6 organelle-like borders of 3 transgenic insertions

Insertion	Border	Sequence type	Length (bp)	Identity with orgDNA (%)	Sunset matches		
					Identity with inserts (%)	Count	Combined length (bp)
**Functional insert:*cp***	A	pt	4000	100.00	97.01	12	4180
	B	pt	1790	99.94	99.09	6	1944
**Nonfunctional insert: pseudo-*nptII***	A	pt	363	100.00	97.96	1	49
	B	pt	827	100.00	93.68	5	1231
**Nonfunctional insert: pseudo-*tetA***	A	pt	6299	98.60	95.09	43	4242
	B	mt	1708	98.18	99.09	2	1738

Notes: chloroplast (pt); mitochondria (mt)

We developed a strategy based on massive paired-end mapping (Fig. 5b) to further investigate whether these organelle-like sequences at both borders of three insertions were present in the genome prior to bombardment or not. If a deletion in ‘Sunset’ relative to the ‘SunUp’ insertion-with-the border region was found (Fig. 5b), we were able to deduce that those organelle-derived fragments flanking transgenic insertions were originally present in ‘Sunset’ prior to bombardment. Deletions were identified using paired ends spanning the specified genomic region in ‘SunUp’ that were longer than the transgenic insert size (*cutoff*).

As a result, a total of 217,890 ‘Sunset’ short reads could be aligned to the region of the functional transgene insertion with organelle-like borders, of which 183,488 reads were mapped to the reference region in properly paired orientations. According to statistical calculations, the inner distances between PE reads were far less than the *cp* functional transgenic insert size (9789 bp), which ranged in size from 0 to 246 bp. Of these, the inner distances of 0 bp were significantly enriched, with 1320 pairs. Meanwhile, there were 42,273 ‘Sunset’ short reads aligned to the region of the nonfunctional *nptII* transgene insert with organelle-like borders, among which 22,518 were mapped as a pair, with pairwise distances ranging from 0 to 169 bp in length. All pairwise distances were smaller than the size of the nonfunctional *nptII* fragment insert at 290 bp. A major fraction of PE reads (223 pairs) were found to have no distance between each other. Regarding the nonfunctional *tetA* transgene insert with both flanks, the total number of mapped reads were 418,697, including 150,110 optimally mapped PE reads. Of the latter, the sizes of inner distances were in the range of 0 to 969 bp, which is under the *cutoff* 1533 bp. There was a significant enrichment for the 0 bp inner distance as well, containing 2661 pairs. The distribution of mapped paired-end spans in regions of three inserts with flanks is shown in histograms (Fig. 5c). Except for the 0 bp distance, three histograms of paired-end inner spans were normally distributed and showed primary peaks at 59 bp (1296 pairs), 57 bp (187 pairs) and 56 bp (1993 pairs). In summary, in all cases of three transgenic insertion events, the inner distance of any pair of mapped PE reads was shorter than a transgenic insert size (*cutoff*), indicating that the distance between any pair of ‘Sunset’-derived PE reads was not elongated by an insertion. This serves as a strong hint that these flanking norgDNAs were not originally contiguous in GM-free Sunset genome.

## Discussion

Conformation of the presence or absence of unintended alterations in addition to target gene integration is a key issue in the evaluation of GM plants. Plant tissue culture processes required during post-transformation can introduce somaclonal variations, which could cause unintended genetic and epigenetic changes leading to heritable phenotypic alterations, as bombardment-mediated transformations can [27]. Besides, since the ‘Sunset’ cultivar we sequenced in the current study was not the progenitor one used to generate ‘SunUp’, spontaneous mutations during 25 generations would also contribute to the divergence between ‘SunUp’ and ‘Sunset’ cultivars.

Revolutionary breakthroughs in NGS in conjunction with developments in bioinformatic software that are tailored to solve biological problems have assisted in the molecular characterization of GM crops and detecting their genome-wide genomic variants induced by somaclonal variations, spontaneous mutations and transformations. For instance, a deep sequencing coverage of 75× in transgenic soybean has uncovered the insertion site of T-DNA [28]. In the case of transgenic rice OSCR11 expressing a seed-based edible vaccine against Japanese cedar pollinosis, 11.3–33.2× whole-genome sequencing was adopted to reveal that the genomic discrepancy between OSCR11 and its host a123 was small, and that nucleotide substitution profiles were analogous to somaclonal variation [29]. Whole-genome sequencing (7×) and CGH arrays were performed to evaluate the molecular composition of herbicide-tolerant mutant rice generated by *Agrobacterium*-mediated gene targeting (GT). In inspected GT rice plants, more than 1000 SNPs and InDels were identified and over 300 somaclonal mutations were predicted to be induced between generations, although no integration of *Agrobacterium*-derived DNA fragments had been detected [30]. The availability of the ‘SunUp’ papaya draft genome, the rapid evolution of deep-sequencing technology along with increasingly robust bioinformatics tools make it possible to decipher genome-wide structural perturbations at the single-base resolution level in the transgenic papaya genome after subjection to bombardment, tissue culture, and other spontaneous mutations during 20-year’s separation.

Here, we carried out paired-end sequencing of DNA-Seq libraries prepared from genomic DNA extracted from young tender healthy leaf tissues of non-transgenic host ‘Sunset’. More than 74 million clean paired-end reads were generated from high throughput DNA sequencing, which translates to an average depth of coverage of 24.72×. The sequencing depths were evenly distributed across the nine papaya chromosomes, indicating a high randomicity performance of Illumina sequencing. After removing multiple mapping reads and PCR duplicates, nearly 100% reads could be uniquely mapped on the ‘SunUp’ reference genome, suggesting a well-assembled reference genome and high levels of similarity between ‘SunUp’ and ‘Sunset’ genome.

### Factors responsible for polymorphisms in ‘SunUp’

In total there were 310,364 SNPs and 34,071 small InDels detected between ‘Sunset’ and the ‘SunUp’ reference genome. Detailed estimates of genetic relationships among papaya accessions and related species were revealed by AFLP makers, suggesting the smallest genetic variation among papaya cultivars derived from the same or similar gene pools [31]. A similar trend has also been observed between distantly related papaya varieties of ‘SunUp’ and nontransgenic ‘AU9’ [32]. Comparative genomic analysis between two homologous BACs from ‘AU9’ and ‘SunUp’ revealed 99% gapless sequence identity, further confirming the limited diversity among papaya varieties by virtue of self-pollination in hermaphrodite papaya and its coexistence and cross-breeding with dioecious varieties. In this study, transgenic papaya ‘SunUp’ was transformed from its nontransgenic progenitor ‘Sunset’, therefore they share genetic similarity with each other, with an average SNP mutation rate of 0.084% in ‘SunUp’, i.e. around 8.4 × 10^− 4^ bases per papaya genome, almost matching the genetic heterozygosity at 0.06% in the ‘SunUp’ genome [33]. This SNP mutation rate is about an order of magnitude greater than the 0.0077% SNP polymorphism rate between the X chromosome and its homologous X^h^ counterpart, but conversely one order lower than the 0.261% SNP rate between recently diverged (< 7 MYA) Y and Y^h^ chromosomes from the same papaya varieties [34].

Compared with other species, this observed SNP rate between ‘SunUp’ and ‘Sunset’ is far lower than those reported from other wild-type plant species [35, 36]. SNP frequency varies from 0.53 to 0.78% between two cultivated rice subspecies *japonica* and *indica* [36]. An overall SNP rate of 0.17% was found between soybean MYMIV (*Mungbean yellow mosaic India virus*) resistant cultivar and susceptible cultivar [35]. In contrast, this SNP rate between ‘SunUp’ and ‘Sunset’ is much higher than the transformation-specific mutation rate and somaclonal mutation rate observed in other species [29, 37, 38]. The pattern of nucleotide base substitution in transgenic rice OSCR11 relative to its nontransgenic host was consistent with somaclonal variation, with a transformation-induced SNP rate of 0.68 × 10^− 7^ per cell culture week [29]. This was highly comparable with the rate induced by somaclonal variations in *Arabidopsis* (0.86 × 10^− 7^) [37] and rice (0.85 × 10^− 7^) [38] per cell culture week.

A previous report indicated that gene mutation rate of transgenic plants was two orders of magnitude less than that observed between soybean cultivars, genetic variants which occurred spontaneously [39]. Given that ‘SunUp’ and ‘Sunset’ papaya have separated for more than 25 years, a theoretical mutagenesis rate in ‘SunUp’ compared with ‘Sunset’ was calculated by dividing the detected mutation rate (8.4 × 10^− 4^) by 25, resulting in a mutation rate of 3.36 × 10^− 5^ per generation. Ossowski et al. [40] reported a spontaneous mutation rate of 6.0 × 10^− 9^ mutations/effective site calculated for *Arabidopsis*. The detected spontaneous mutation rate in papaya off-type SSR markers was rather high at 3% frequency after one meiosis [41]. Considering the transformation-induced SNPs in ‘SunUp’ cannot readily be distinguished from somaclonal and spontaneous variants, and the mutation rate induced by transformation and somaclonal variations was far less than the spontaneous mutation rate, we speculate that ongoing spontaneous mutations induced through propagation and regeneration during 25 years of separation is a primary mutation type in particle bombardment-mediated transformed ‘SunUp’. The genetic variants accumulated through ongoing spontaneous mutation over numerous generations were not found to pose any new risk to consumers, as they likely already evolved through natural selection [39].

Both SNPs and small InDels were randomly produced amongst papaya chromosomes (Table 3), indicating that biolistic based transformation could have a genome-wide effect on the papaya genome, not just specifically affecting the flanking sequences of insertion sites. Interestingly, an uneven distribution was observed for those mutations with the highest density in chromosome 6 and the lowest in chromosome 2. Although sequences of three ‘SunUp’ transformation plasmid vector derived inserts with genomic borders had been well characterized [7], their exact genomic positions in ‘SunUp’ remain enigmatic owing to technical limitation [42]. The increased levels of nucleotide variation in chromosome 6 imply this chromosome might experience strong disturbances in the genomic stability accompanied by transgene integration. As reported by Doerfler et al. [43] exogenous DNA insertion can have genome-wide perturbations that are not limited to the insertion site, and possibly transmitted to neighboring DNA sequences, to chromatin structures and even to adjacent chromosomes that are in contact with the insertion site of the chromosome targeted by foreign DNA insertion. Multiple insertions separated by genomic DNA in one single chromosome were reported to be a common occurrence in biolistic based transformation [44, 45]. We surmise that three transgenic inserts were likely inserted in one chromosome. This conjecture remains to be further studied. Additionally, sources of bias and error, such as technical variability during library preparation and sequencing, sequencing bias and the inevitable error rates during short read alignment in highly repetitive regions especially in repeat-rich gene-poor heterochromatin, may account for the relative high frequency of mutations in some regions.

In terms of base substitution type, bias towards G/C to A/T transitions was observed in this study (Table 4, Fig. S2). This result support previous reports on the pattern of nucleotide substitutions, regardless of whether SNPs were caused by spontaneous and somaclonal mutations [29, 40], chemical and physical mutagens [46], or by *Agrobacterium*-mediated gene targeting and transformation [30, 47]. Overabundance of G/C to A/T fit the earlier theory that G:C sites in CpG contexts are more likely to be methylated [48], and spontaneous deamination of methylated cytosine would lead to thymine substitution [49, 50].

However, non-methylated G:C sites also had a higher rate of transition than A:T sites in *A. thaliana*, suggesting that other factors in addition to methylation are responsible for the high rate of transitions at G:C sites [40]. Other studies have shown that G/C to A/T transitions frequently happen at dipyrimidine sites where the C is adjacent to another C or to a T under ultraviolet (UV) radiation, which exists in natural conditions [51]. Another supporting theory was proposed that alkylated guanines are easily paired with thymines, leading to misplaced adenines at sites of guanines [52].

The combined effect of the deamination of methylated cytosines, alkylated guanines and UV-induced mutagenesis could explain the increased rate of transitions at G:C sites in our study. The determined Ts/Tv ratio was 1.95, which is comparable to ratios of 1.9781 and 1.9609 found in soybean MYMIV susceptible and resistant cultivars [35], lower than the 2.4 ~ 2.7 ratio reported for spontaneous mutations in *Arabidopsis* mutation accumulation lines [40], but obviously higher than the Ts/Tv ratios in transformation-induced SNPs or somaclonal variation induced SNPs of nearly 1.0 [37, 38]. Transitions are interchanges of two-ring purines (A/G) or of one-ring pyrimidines (C/T), and can be generated at higher frequency than transversions under natural conditions. Transversions, on the contrary, are reported to be more common when subjected to high levels of genetic instability [53]. G/C to T/A transversions arise because oxidized guanines (8-hydroxy-G) are prone to pairing with adenines instead of cytosines and leading to misplaced thymines in the positions where guanines should be [54]. We conclude that the increased Ts/Tv ratio in our study, relative to transformation-induced and somaclonal variation, can be explained by the coupled effects of genetic transformation, somaclonal and spontaneous variants, but largely caused by spontaneous variants.

We observed an excess of 1 to 2 bp-sized InDels and a significant deficit of 5 bp-sized InDels (Fig. 1). Small InDels preferentially occurred in repetitive regions such as microsatellites and homopolymers [37], the mutational nature of which was mainly attributed to the DNA replication slipped-strand mispairing (SSM) mechanism [55, 56]. In agreement with previous studies, nearly all InDels directly engendered by transformation and somaclonal mutation were 1 or 2 bp in size except for one 5-bp transformation-induced deletion [29, 37]. All of the 1- or 2-bp InDels occurred in a mono- or di-nucleotide context as a result of slippage during DNA replication. Since the exceptional 5-bp deletion was in a non-polymeric context, it may be attributable to the improper repair of a DNA double strand break (DSB) caused by transformation or it could have happened spontaneously.

Based on SNPEFF results, most SNPs and InDels were detected in intergenic regions. As compared with genic regions, SNPs and InDels were much denser in the upstream and downstream regulatory regions (Fig. 2). Non-coding regulatory regions of genes contained lower level of sequence conservation and purifying selection pressure relative to coding regions, which could account for the enrichment of variations in the upstream and downstream regulatory regions [57]. The NS/Syn ratio was 1.439, and a collection of 1454 high-impact genes affected by SNPs were predicted (Table 5). Functional annotation suggested that ATP catabolic process and ribonucleotide catabolic process were highly enriched among these high-impact genes. Information on those high-impact mutations would be useful for the development of DNA markers associated with disease-resistance related genes, which could advance marker-assisted disease resistance breeding in papaya.

Following manual inspection of read alignments by IGV software, all SVs were identified as false positives largely owing to the incompleteness of the papaya genome and the limitation when using Illumina short reads. A more completely assembled and gapless papaya reference genome together with long sequencing reads are needed in the future for dissecting large structural variations. Previous reports analyzing somaclonal variations in *Arabidopsis* and rice showed that no SVs were detected [37, 38], we surmise that large SVs were likely caused by integration position effects of particle bombardment transformation.

### NorgDNAs flanking the inserts as a result of the transformation

Organelle-to-nucleus DNA transfers are continually ongoing in plant genomes [18, 58]. We developed two pipelines for the automatic identification of norgDNA junction sites in ‘SunUp’ and ‘Sunset’ in this study, and results showed that altogether 3327 NUPT and 2642 NUMT junction sites were shared by ‘SunUp’ and ‘Sunset’, covering at least 95% of total norgDNA junction sites. Our data provide direct evidence that norgDNAs are widely spread throughout the papaya genome and are highly conserved between the transgenic papaya ‘SunUp’ and its nontransgenic precedent cultivar ‘Sunset’. It can also be inferred from the high conservation that the vast majority of norgDNAs were older transfers predating the transgenic event and sparse organelle-to-nucleus integrations were triggered by transgenes. Those ancient norgDNAs might play a critical role in papaya genome evolution. This result agrees with earlier findings, which shows that newly formed norgDNAs tend to be fragmented, shuffled and rapidly eliminated [14, 59]. The transfer amount and rates of pt. and mtDNA in the nucleus differs among species. The accumulation of norgDNAs is driven by selective pressure or recombination suppression and norgDNAs would accumulate to a varying extent even in different regions of the same genome. As previously reported [13], the lack of recombination in papaya HSY and MSY regions made them accumulate 4 times the amount of NUPTs than the papaya whole genome average and almost 12 times than the corresponding region of the X chromosome. By contrast, NUMTs were less prevalent in the X and HSY chromosomes compared to the genome-wide distribution. Furthermore, only a few norgDNAs were conserved between X and HSY, indicating that the accelerated accumulation of norgDNAs occurred after the recombination suppression in the HSY.

Those ‘Sunset’ or ‘SunUp’ regions where specific norgDNAs are detected could be newly formed via shuffling and the rearrangement of extant genomic norgDNA fragments when bombardment-induced exogenous DNA was integrated into the genome causing instability, or new transfer from organelle genomes which was accompanied by bombardment-mediated transformation. Older inserts from organelles are predicted to exhibit lower pt./mt DNA identities due to fragmentation and mutation that occurs over time [18, 60]. As well characterized in *Oryza* and *Arabidopsis* [61], clusters of NUPTs and NUMTs contained in the angiosperm nuclear genomes can be very fragmented and rearranged with respect to the extant organelle genomes. Hence, the evolutionary change of individual norgDNA fragments since integration into the nuclear genome can be estimated by comparison with organelle genomes in this current analysis. The variable matches to papaya organelle genomes indicate that the fragments transfer in different periods, with some predating the bombardment and others taking place within the last 25 years. We also found that the average identity between ‘SunUp’-specific norgDNAs and the extant organelle genomes was higher than that of conserved norgDNAs between ‘SunUp’ and ‘Sunset’ (Table 6). It can be inferred that biolistic based gene transformation could accelerate the DNA transfer frequency and amount from organelles into the papaya nuclear genome, and that new organelle-to-nucleus DNA integration probably occurred during bombardment.

Three transgenic inserts in ‘SunUp’ are surprisingly flanked by norgDNA segments, with five NUPTs and one NUMT. The higher ratio of NUPT:NUMT (5:1) is expected because it is proportionally close to the ratio of pt.:mt genome (5.5:1) in the cell. The average read depths from the whole genome shotgun reads for the pt. and mt genomes are 1044 and 189 respectively (data not shown). This predicts a pt.:mt genome ratio of 5.5. NUPTs were observed to be more abundant than NUMTs on the genome-wide scale as well, according to our findings (Table 6). The distribution of norgDNAs showed a similar trend to SNPs, in that they are overrepresented in Chr6 compared to other chromosomes. This finding further implies that Chr6 may experience strong perturbations in genome structure in the event of foreign DNA being inserted. To estimate whether those organelle-like border fragments were present in the genome prior to bombardment or not, we initially examined the identities between six ‘SunUp’ organelle-like borders and papaya organelle genomes. All six border sequences, especially plastid-like borders, were nearly identical to the corresponding sequences in the extant papaya organelle genomes (98.18 ~ 100%), this being significantly higher than the identity of conserved norgDNAs compared with organelle genomes (91.92 ~ 92.97%). Six organelle-like borders with high nucleotide identity relative to organelle genomes likely represent newer transfers of DNA. Homology searches between norgDNAs in ‘Sunset’ and six organelle-like borders in ‘SunUp’ in the follow-up step showed that all five NUPT borders had relatively lower similarities to ‘Sunset’ norgDNAs (93.68 ~ 99.09%) than to the papaya pt. genome (nearly 100%) (Table 8), demonstrating that new transfers of DNA from chloroplast to the nuclear genome occurred including five plastid-like borders following bombardment-induced foreign gene insertion. We did not expect that the NUMT border would match ‘Sunset’ norgDNAs with a slightly higher similarity (99.09%) than to the mt genome (98.18%). Based on the massive paired-end mapping strategy, we did not find the inner distance of mapped ‘Sunset’ PE reads was elongated by a transgenic insert. This further confirmed that six organelle-like border sequences were not present in the recipient genome antecedent to particle bombardment-mediated transformation, and it is likely that they were newly added to the papaya nuclear genome from organelles in the wake of gene transfer although the integration mechanism underlying bombardment-induced norgDNA remains to be elucidated. Two hypotheses were put forward in this study. One hypothesis is that the acquisition of many bases of inserted DNA increased the instability of the papaya genome and likely altered the chromatin topology, enabling organelle DNA fragments to be readily integrated into the nuclear genome. When encountered DNA lesion such as DNA double-strand breaks triggered by exogenous sequences, cells respond by activating a DSB repair mechanism [62]. Accumulating evidence indicates that most norgDNAs integrate into the nuclear genome via a non-homologous recombination or NHEJ-DSB repair mechanism as any other exogenous sequences [18]. Another possibility is that foreign genes initially insert into the chloroplast genome are spontaneously shifted into the nucleus with sections of adjacent chloroplast DNA. Several different studies have shown that a plastid transgene *nptII* was successfully transferred into the nuclear genome from the plastid, which was found to happen at a surprisingly high frequency of approximately one in five million cells [60, 63]. The foreign DNA tends to integrate randomly into the host genome via biolistic based transformation, so it is not possible to determine where the transgene initially inserted and evidence in support of this assumption is largely lacking. We hope that further studies based on our results will lead to remarkable breakthroughs in the filed of plant genetic engineering.

## Conclusions

The main target of this research was to thoroughly inspect genome-wide discrepancies between the PRSV resistant transgenic papaya ‘SunUp’ and its nontransgenic progenitor cultivar ‘Sunset’, including small SNPs/InDels, large SVs, and nuclear organelle DNA integrations. Detected variations were randomly distributed amongst papaya chromosomes, whereas only 0.27% were predicted to have a disruptive impact on the protein function. Development of SNP/InDel markers that occurred in high-impact genes could facilitate marker-assisted PRSV disease resistance breeding in papaya. Genome-wide analysis of organelle-to-nucleus integration events confirmed that norgDNAs are ubiquitous in papaya genome and highly conserved before and after genetic transformation. Those conserved norgDNAs might play a pivotal role in papaya nuclear genome. We reasoned that biolistic transformation could speed up the organelle-to-nucleus transfer frequency and amount, and six organelle-like borders of transgenic inserts likely newly transferred to the nucleus in the wake of bombardment-induced foreign gene insertion. The newly integrated norgDNA induced by particle bombardment revealed the mechanisms underlying the process of foreign gene transformation. The major cause of polymorphisms in ‘SunUp’ is likely to be spontaneous mutation. Therefore, any speculated risk due to the unintended consequences of biolistic transformation in ‘SunUp’ should only merit the same consideration given to variations arising spontaneously from traditional breeding practices, which attests to the safety of transformation technology. A completely assembled papaya genome in the near future will complement the present study.

## Methods

### Plant material and next-generation sequencing

The non-transgenic progenitor papaya cultivar ‘Sunset’ plants were grown under natural conditions at Kunia substation in Oahu, Hawaii by Hawaii Agriculture Research Center. The soil moisture and plants were checked daily and watered as needed. These ‘Sunset’ plants were not the progenitor line used to generate transgenic SunUp, but have already grown independently for over 25 generations after transformation event. Three months after planted, young and healthy leaf tissues from a best-growing individual were collected for DNA extraction. Total genomic DNA was extracted from leaves using a modified approach for reduced organelle contamination [64]. The NanoDrop 2000 spectrophotometer (Nano-Drop Technologies, USA) was applied to evaluate the concentration and quality of DNA. The isolated DNA were measured with an average OD (260/280) close to 1.8 and the concentration of per DNA sample should be more than 100 ng/μl. The frozen samples including leaf tissues and genomic DNA were preserved in Ming’s laboratory in University of Illinois at Urbana-Champaign (UIUC) and can be acquired with the voucher number Sunset-A07.

Sequencing of papaya ‘Sunset’ genome was carried out at the W. M. Keck Center for Comparative and Functional Genomics, UIUC. A paired-end DNA library was constructed with an insertion size of 250 bp and subjected to Illumina DNA short reads sequencing on Illumina HiSeq2000 platform (Illumina Inc., San Diego, CA, USA). Over 74 million 124 bp paired-end reads were generated from one lane of sequencing. Prior to any downstream processing, the empty reads, poor quality reads and adaptor sequences in the raw sequenced data (> 30% of the bases with a Phred quality score of <Q20) were filtered out using the program IlluQC.pl in NGSQCToolkit [65] to obtain clean reads (Fig. 3a). Graphs showing QC statistics were generated. After filtering, the NGSQCToolkit was used to check the data quality again.

### Genome-wide detection of SNPs, small InDels and large structural variants

Sequences of three ‘SunUp’ transformation plasmid derived inserts with genomic borders could not be assembled into the ‘SunUp’ genome (Genbank accession number: GCA_000150535.1) which was ascribed to technical limitations, therefore three inserts and their flanking sequences were not taken into account in the genome-wide detection of SNPs, InDels and SVs. In order to further detect other plasmid vector derived inserts in ‘SunUp’ reference genome in addition to the three aforementioned well-known plasmid-derived inserts, the BLASTN [25] program was conducted to search with the entire transformation plasmid (19,567 bp) as a nucleotide query against the whole ‘SunUp’ reference genome as a database.

The resulting paired-end reads of ‘Sunset’ were aligned to the most updated ‘SunUp’ genome by BWA’s short read aligner with default parameters [21]. Only uniquely mapped reads were retained by choosing the “@SQ|@PG|@RG|XT:A:U” tag in raw output Sequence Alignments/Map (SAM) format file to ensure that a read only had a single mapped location. The unimap SAM file was then converted to Binary Alignment/Map (BAM) format, sorted according to chromosomal coordinates, treated for potential PCR duplicates removal and indexed using the SAMtools software suite [22]. The SAMtools ‘mpileup’ utility was performed to call SNP variants with -ugDV parameters followed by ‘bcftools’ from the SAMtools package. Polymorphism information was stored in a variant call format (VCF) file. The raw variant calls were filtered with the SAMtools vcfutils.pl varFilter script and a custom script vcf_filter.py for read depth ≥ 10 and ≤ 100 and polymorphism site quality ≥50. An SNP site at which two or more alternate alleles (ALT) were called was removed for diploid organisms.

Variant effect analysis of SNPs and InDels were predicted on the basis of information on gene structure and function in papaya using SNPEff (ver. 4.1) [23]. Since papaya genomic annotation database is not available in the pre-built databases of SNPEff, we built a database for papaya using the ‘SunUp’ reference genome in FASTA format and its gene annotation file in GFF format. The potential effect of each variant on gene expression and protein structure or function was examined by SNPEff. GO terms describing the biological processes, molecular functions and cellular components were assigned to the high-impact genes using the Blast2GO program [24]. Further, GO enrichment analysis for high-impact genes was performed in the agriGO program [66] with Fisher statistical test and Bonferroni’s correction (FDR ≤0.05). The gene models of papaya ‘SunUp’ reference genome were used as a background. BreakDancer [67] was used to detect genomic SVs using ‘Sunset’ read pairs that are mapped to ‘SunUp’ reference genome with unexpected separation distances or orientations. BreakDancer predicts five types of structural variants: insertions (INS), deletions (DEL), inversions (INV), inter- and intra-chromosomal translocations (ITX and CTX). The SVs were filtered by scores equal to 99 and number of reads ≥10 thereby selecting a highly confident set of SVs.

### ‘SunUp’-specific nuclear organelle DNA junction sites

The BLASTN [25] algorithm was used to search the ‘SunUp’ genome for nuclear plastid (NUPT) and nuclear mitochondria (NUMT) integrations with papaya (*Carica papaya*) organelle genomes as databases (Fig. 3b). The organelle genomes are available at Genbank: the chloroplast and mitochondria genome under accession number EU431223 and EU431224, respectively. An *E*-value cut-off of 1e-20 with > 80% homology is included in the analyses.

A set of clean single-end reads of ‘Sunset’ were aligned against the ‘SunUp’ reference genome using BWA v0.7.12 with default settings (Fig. 3c). After the alignment, the mixture of reads that aligned back to the reference genome were predicted to originate from different sources of DNA in ‘Sunset’ genome, including nuclear DNA (nuDNA), nuclear organelle DNA (norgDNA) and organelle DNA (orgDNA) (Fig. 3e). We labeled the joint position that lies at the junction of norgDNA and nuDNA as a junction site. Only a junction site in the ‘SunUp’ reference genome that was both mapped and spanned by ‘Sunset’ reads can be termed a shared junction site, and it is considered to be shared by both ‘SunUp’ and ‘Sunset’ genomes (Fig. 3d). In order to discriminate between these three categories of reads and obtain the reliable junction sites shared by ‘SunUp’ and ‘Sunset’, the flanking regions (5 bp upstream and downstream) of the junction sites are used as an indicator. Reliable norgDNA reads were selected if those reads were not only spanning the junction sites but also mapped at least to 5 bp of norgDNA or nuDNA (Fig. 3e). Otherwise, if there were no reads mapped to or no reliable norgDNA reads spanning the junction site, we considered this junction site a ‘SunUp’-specific norgDNA junction site (Fig. 3f). An in-house software pipeline written in a mixture of python scripts (available upon request) was developed for automatically processing and identifying norgDNA junction sites in ‘SunUp’. This pipeline is well documented and widely applicable to other diploid plants. We manually visualized the alignment output in the Integrative Genomics Viewer (IGV) software [26] and ensure the validity and reliability of those ‘SunUp’-specific norgDNA junction sites identified by this pipeline.

### ‘Sunset’-specific nuclear organelle DNA junction sites

We aligned a set of clean single-end reads of ‘Sunset’ to chloroplast and mitochondria as reference genome independently using Bowtie2 version 2.2.5 [68] (Fig. 4a). The CIGAR (Compact Idiosyncratic Gapped Alignment Report) strings of reads which represent the sequence alignment in SAM/BAM file were used to identify and extract soft-clipped reads with at least 5 bp mismatches at the extremity (Fig. 4b). Those reads were de novo assembled by SOAPdenovo 63 mer-V2.04 [69] with an optimized *K*-mer length of 63 to generate potential norgContigs that were as long as possible (Fig. 4c). The norgContigs were screened for sequence similarity by BLAST against corresponding organelle genome at an *E*-value cut-off of 1e-20 (Fig. 4d). Only hits of norgContigs with ≥30 bp mapped to organelle genomes and ≥ 5 bp unmatched on the edge were considered as reliable norgContigs and used in further study. The ‘Sunset’-specific norgDNAs were obtained when no hits were determined by BLAST against ‘SunUp’ reference genome (*E*-value ≤1e-5) (Fig. 4e). An in-house software pipeline written in a mixture of python scripts (available upon request) was developed for automatically processing and identifying of norgDNA junction sites in ‘Sunset’.

### Identity between ‘SunUp’ organelle-like Borders of transgenic inserts and ‘sunset’ NorgDNA

All three sequences of ‘SunUp’ transformation plasmid derived inserts with borders are available at Genbank under accession numbers FJ467933, FJ467932 and FJ467934, respectively (Fig. 5a). Six organelle-like sequences flanking three ‘SunUp’ transgenic insertions could be extracted from them. Those organelle-like borders were screened for organelle genome similarity using BLAST (*E*-value ≤1e-5).

In order to see whether these six organelle-like border sequences were present in the genome prior to bombardment or not, we set out to examine the identity between norgDNAs in ‘Sunset’ and the flanking norgDNAs of inserts in ‘SunUp’. To see the identity between them, searches between reliable ‘Sunset’ norgContigs as query against two organelle genomes and six organelle-like borders as databases were separately performed with BLASTN using an *E*-value cut-off of 1e-5 (Fig. 4f). Blast hits between ‘Sunset’ norgDNA and ‘SunUp’ organelle-like borders were considered the best hits if the query-start and query-end of one hit in blast-output2 matches the hit with the same query ID in blast-output1. The other option to be considered a best hit would be if the longest hit (which cannot be any longer and is shorter than norgDNA in corresponding hit in blast-output1) of one query ID in blast-output2 totally matches the corresponding part of hit in blast-output1.

### Identification of the origin of ‘SunUp’ organelle-like Borders of transgenic inserts

In order to see whether these six organelle-like border sequences were present in the genome prior to bombardment or not, we developed a strategy which utilizes high-throughput and massive paired-end mapping to identify deletions in ‘Sunset’ relative to the reference genome (Fig. 5). Clean paired-end reads of ‘Sunset’ were aligned against three ‘SunUp’ transformation plasmid derived inserts with borders as a whole by using BWA’s short read aligner with default parameters. After removing multiple mapping reads, the unimap alignments were converted from SAM format into BAM format. Aligned reads were then sorted, treated for potential PCR duplicates removal and indexed using SAMtools. Three BAM files of read alignments in regions of three inserts with borders could be separated according to reference names using SAMtools ‘view’ command. Name-sorted BAM files were converted to BED with bamToBed script from the BEDTools package [70]. A deletion in ‘Sunset’ relative to the reference genome was identified using paired-end reads spanning the transgenic insert region. If the inner distance of paired-end reads in the reference genome was longer than a transgenic insert size then a deletion had taken place. If this was found to be the case, the flanking norgDNA of transgenic inserts in transgenic cultivar ‘SunUp’ were identified as native to its progenitor ‘Sunset’. Histogram plots of the inner distance of mapped paired-end reads in regions of three inserts with borders could be generated by the R version 3.2.1 statistical package (www.CRAN.R-project.org).

## Supplementary information

**Additional file 1: Table S1.** Distribution of structural variations on nine pseudomolecules of papaya.

**Additional file 2: Table S2.** GO enrichment analysis for the putative high mutation genes.

**Additional file 3: Table S3.** The genome positions of specific chloroplast DNA integration sites in SunUp.

**Additional file 4: Table S4.** The genome positions of specific mitochondrion DNA integration sites in SunUp.

**Additional file 5: Figure S1.** Chromosomal and genome-wide distribution of the frequency of A. SNP/InDels B. SNPs and C. InDels per 1 kb in the Sunset genome compared to the SunUp reference genome.

**Additional file 6: Figure S2.** Transitions and transversions of homo and hetero SNPs identified in the Sunset genome compared to the SunUp reference genome. **A.** Frequency of different nucleotide substitution types in homo and hetero SNPs. **B.** Numbers and percentages of transitions (Ts) and transversions (Tv), and the Ts/Tv ratio in homo/hetero SNPs and total SNPs.

**Additional file 7: Figure S3.** AgriGO results of putative high mutation genes based on SEA analysis. Significance level of enrichment is displayed using a color scale. White indicates no significant enrichment; color transitions from yellow to red indicate an increase in the strength of significance. Ratios at the bottom of each GO box represent the number of genes in the input list matching that GO term versus the number of total genes in the input list, total genes in the background genome set matching that GO term versus total genes in the background set. The adjusted *p*-value for each enriched GO term is indicated in parentheses at the top of each colored box.

## Data Availability

The Illumina DNA-sequencing raw reads of nontransgenic cultivar ‘Sunset’ have been deposited in the National Center for Biotechnology Information (NCBI) Sequence Read Archive database (SRA; https://www.ncbi.nlm.nih.gov/sra/) under project accession number of PRJNA578028. The pipelines used during the current study are available from the corresponding author on reasonable request. The ‘SunUp’ reference genome and annotation file were obtained from NCBI Genbank with accession number GCA_000150535.1. The papaya chloroplast and mitochondria genome sequences were obtained from NCBI Genbank with accession number EU431223 and EU431224, respectively.

## Abbreviations

ALT: Alternate alleles
array-CGH: Comparative genome hybridization
BAM: Binary Alignment/Map
CDS: Coding sequence
*cp*: Coat protein
CTX: Inter-chromosomal translocations
DEL: Deletions
DSB: DNA double strand break
GM: Genetically modified
GO: Gene Ontology
IGV: Integrative Genomics Viewer
InDels: Small Inserts/deletions
INS: Insertions
INV: Inversions
ITX: Intra-chromosomal translocations
mt: Mitochondria
MYMIV: *Mungbean yellow mosaic India virus*
NGS: Next-generation sequencing
NHEJ-DSB repair: Non-homologous end joining of double-strand break repair
norgDNA: Nuclear organelle DNA
NS/Syn: Non-synonymous to synonymous
nuDNA: Nuclear DNA
NUMT: Nuclear mitochondrial DNA
NUPT: Nuclear plastid DNA
orgDNA: Organelle DNA
PDR: Pathogen-derived resistance
PE: Paired-end
PRSV: *Papaya Ringspot Virus*
pt.: Chloroplast
SAM: Sequence Alignments/Map
SNPs: Single nucleotide polymorphisms
SSM: Slipped-strand mispairing
SVs: Structural variations
Ts: Transitions
Tv: Transversions
UV: Ultraviolet
VCF: Variant call format
