# Supplementary material for: Genomic variation between PRSV resistant transgenic SunUp and its progenitor cultivar Sunset
Source: BMC Genomics. 2020 Jun 12;21:398. doi: 10.1186/s12864-020-06804-7 (PMC7291442; doi:10.1186/s12864-020-06804-7)
Supplement: Supplementary file 1 — Additional file 1: Table S1. Distribution of structural variations on nine pseudomolecules of papaya. [file 12864_2020_6804_MOESM1_ESM.docx]

Additional file 1: Table S1. Distribution of structural variations on nine pseudomolecules of papaya

| **Type of SVs^1^** | **Chromosomes** | |  |  |  |  |  |  |  | **Subtotal** |  |  | **Total** |
| --- | --- | --- | --- | --- | --- | --- | --- | --- | --- | --- | --- | --- | --- |
|  | **chr1** | **chr2** | **chr3** | **chr4** | **chr5** | **chr6** | **chr7** | **chr8** | **chr9** |  | **unanchored^3^** | **uncertain^4^** |  |
| CTX | 5 | 8 | 0 | 3 | 7 | 11 | 7 | 6 | 11 | 58 |  | 891 | 949 |
| ITX^2^ | 2 | 7 | 7 | 1 | 0 | 3 | 1 | 2 | 4 | 27 | 17 |  | 44 |
| INV | 1 | 0 | 0 | 0 | 0 | 1 | 0 | 0 | 1 | 3 | 0 |  | 3 |
| INS | 5 | 13 | 6 | 2 | 4 | 11 | 5 | 1 | 1 | 48 | 28 |  | 76 |
| DEL | 9 | 14 | 10 | 8 | 13 | 14 | 3 | 5 | 8 | 84 | 44 |  | 128 |
| Total | 22 | 42 | 23 | 14 | 24 | 40 | 16 | 14 | 25 | 220 | 89 | 891 | 1200 |

Notes:

^1^ CTX: inter-chromosomal translocations; ITX: intra-chromosomal translocations; INV: inversions; INS: insertions; DEL: deletions.

^2^ ITXs detected by BreakDancer only consider intra-chromosomal translocation events that occurred between two non-adjacent portions of one scaffold, we further detected more ITXs manually when an intra-chromosomal translocation happened between a non-adjacent scaffold pair from the same chromosome.

^3^ Scaffolds which have not been anchored to chromosomes to date.

^4^ Those inter-chromosomal translocations occurred between scaffold pairs but one or two of these scaffold pairs have not been anchored to chromosomes.
