# Supplementary material for: Genomic variation between PRSV resistant transgenic SunUp and its progenitor cultivar Sunset
Source: BMC Genomics. 2020 Jun 12;21:398. doi: 10.1186/s12864-020-06804-7 (PMC7291442; doi:10.1186/s12864-020-06804-7)
Supplement: Supplementary file 2 — Additional file 2: Table S2. GO enrichment analysis for the putative high mutation genes. [file 12864_2020_6804_MOESM2_ESM.docx]

Additional file 2: Table S2. GO enrichment analysis for the putative high mutation genes

| **GO term** | **Ontology** | **Description** | **Number in input list** | **Number in BG/Ref** | ***p*-value** | **FDR** | **-log_10_ FDR** |
| --- | --- | --- | --- | --- | --- | --- | --- |
| GO:0006200 | P | ATP catabolic process | 36 | 267 | 1.90E-06 | 0.0011 | 2.958607315 |
| GO:0009261 | P | ribonucleotide catabolic process | 43 | 373 | 1.30E-05 | 0.0072 | 2.142667504 |
| GO:0006195 | P | purine nucleotide catabolic process | 43 | 373 | 1.30E-05 | 0.0072 | 2.142667504 |
| GO:0009146 | P | purine nucleoside triphosphate catabolic process | 43 | 373 | 1.30E-05 | 0.0072 | 2.142667504 |
| GO:0009203 | P | ribonucleoside triphosphate catabolic process | 43 | 373 | 1.30E-05 | 0.0072 | 2.142667504 |
| GO:0009207 | P | purine ribonucleoside triphosphate catabolic process | 43 | 373 | 1.30E-05 | 0.0072 | 2.142667504 |
| GO:0009154 | P | purine ribonucleotide catabolic process | 43 | 373 | 1.30E-05 | 0.0072 | 2.142667504 |
| GO:0009143 | P | nucleoside triphosphate catabolic process | 43 | 374 | 1.40E-05 | 0.0077 | 2.113509275 |
| GO:0009150 | P | purine ribonucleotide metabolic process | 47 | 423 | 1.40E-05 | 0.0079 | 2.102372909 |
| GO:0009166 | P | nucleotide catabolic process | 43 | 375 | 1.40E-05 | 0.0082 | 2.086186148 |
| GO:0006163 | P | purine nucleotide metabolic process | 48 | 436 | 1.50E-05 | 0.0082 | 2.086186148 |
| GO:0046034 | P | ATP metabolic process | 36 | 294 | 1.60E-05 | 0.0093 | 2.031517051 |
| GO:0009144 | P | purine nucleoside triphosphate metabolic process | 44 | 404 | 4.00E-05 | 0.023 | 1.638272164 |
| GO:0009205 | P | purine ribonucleoside triphosphate metabolic process | 44 | 404 | 4.00E-05 | 0.023 | 1.638272164 |
| GO:0009199 | P | ribonucleoside triphosphate metabolic process | 44 | 411 | 6.00E-05 | 0.034 | 1.468521083 |
| GO:0009141 | P | nucleoside triphosphate metabolic process | 44 | 413 | 6.70E-05 | 0.038 | 1.420216403 |
| GO:0017111 | F | nucleoside-triphosphatase activity | 67 | 618 | 7.40E-07 | 0.00011 | 3.958607315 |
| GO:0016818 | F | hydrolase activity, acting on acid anhydrides, in phosphorus-containing anhydrides | 69 | 649 | 1.00E-06 | 0.00016 | 3.795880017 |
| **Table S2. (Continued)** |  |  |  |  |  |  |  |
| **GO term** | **Ontology** | **Description** | **Number in input list** | **Number in BG/Ref** | ***p*-value** | **FDR** | **-log_10_ FDR** |
| GO:0016887 | F | ATPase activity | 44 | 350 | 1.10E-06 | 0.00017 | 3.769551079 |
| GO:0016462 | F | pyrophosphatase activity | 68 | 639 | 1.20E-06 | 0.00018 | 3.744727495 |
| GO:0016817 | F | hydrolase activity, acting on acid anhydrides | 69 | 659 | 1.80E-06 | 0.00027 | 3.568636236 |
| GO:0000166 | F | nucleotide binding | 189 | 2449 | 6.70E-05 | 0.01 | 2 |
| GO:0005524 | F | ATP binding | 131 | 1615 | 8.50E-05 | 0.013 | 1.886056648 |
| GO:0032559 | F | adenyl ribonucleotide binding | 132 | 1632 | 9.10E-05 | 0.014 | 1.853871964 |
| GO:0030554 | F | adenyl nucleotide binding | 141 | 1763 | 9.30E-05 | 0.014 | 1.853871964 |
| GO:0001883 | F | purine nucleoside binding | 141 | 1764 | 9.50E-05 | 0.014 | 1.853871964 |
| GO:0001882 | F | nucleoside binding | 141 | 1774 | 0.00012 | 0.018 | 1.744727495 |
| GO:0032555 | F | purine ribonucleotide binding | 144 | 1821 | 0.00013 | 0.02 | 1.698970004 |
| GO:0032553 | F | ribonucleotide binding | 144 | 1821 | 0.00013 | 0.02 | 1.698970004 |
| GO:0017076 | F | purine nucleotide binding | 153 | 1953 | 0.00013 | 0.02 | 1.698970004 |
| GO:0042623 | F | ATPase activity, coupled | 31 | 274 | 0.00024 | 0.037 | 1.431798276 |
