# Supplementary material for: Genomic variation between PRSV resistant transgenic SunUp and its progenitor cultivar Sunset
Source: BMC Genomics. 2020 Jun 12;21:398. doi: 10.1186/s12864-020-06804-7 (PMC7291442; doi:10.1186/s12864-020-06804-7)
Supplement: Supplementary file 3 — Additional file 3: Table S3. The genome positions of specific chloroplast DNA integration sites in SunUp. [file 12864_2020_6804_MOESM3_ESM.docx]

Additional file 3: Table S3. The genome positions of specific chloroplast DNA integration sites in SunUp

| **Type** | **Border Number** | **Scaffold** | **Insert Position** | |  | **Position in orgDNA** | | **E-value** | **Identity** | **Chromosome** |
| --- | --- | --- | --- | --- | --- | --- | --- | --- | --- | --- |
|  |  |  | **Start** | **End** |  | **Start** | **End** |  |  |  |
| reliable | one border found | supercontig_0 | 2534921 | 2535002 |  | 65309 | 65391 | 2.00E-26 | 95.18 | 6 |
|  | one border found | supercontig_0 | 4570284 | 4571406 |  | 61413 | 62538 | 0 | 95.47 | 6 |
|  | one border found | supercontig_1 | 4849106 | 4849367 |  | 79343 | 79082 | 1.00E-135 | 98.47 | 2 |
|  | one border found | supercontig_2 | 1165138 | 1167532 |  | 149369 | 146974 | 0 | 94.5 | 2 |
|  | one border found | supercontig_2 | 1165138 | 1167532 |  | 99481 | 101876 | 0 | 94.5 | 2 |
|  | one border found | supercontig_2 | 1167600 | 1168987 |  | 102459 | 103848 | 0 | 94.25 | 2 |
|  | one border found | supercontig_2 | 1167600 | 1168987 |  | 146391 | 145002 | 0 | 94.25 | 2 |
|  | one border found | supercontig_2 | 1986365 | 1987213 |  | 56896 | 57744 | 0 | 99.76 | 2 |
|  | both borders found | supercontig_4 | 2283181 | 2283317 |  | 131410 | 131545 | 3.00E-61 | 97.81 | 9 |
|  | one border found | supercontig_5 | 1539355 | 1539605 |  | 104119 | 104369 | 5.00E-127 | 98.01 | 8 |
|  | one border found | supercontig_5 | 1539355 | 1539605 |  | 144731 | 144481 | 5.00E-127 | 98.01 | 8 |
|  | one border found | supercontig_6 | 1946612 | 1946853 |  | 62078 | 62320 | 5.00E-53 | 86.42 | 4 |
|  | one border found | supercontig_6 | 2218545 | 2218805 |  | 37107 | 36847 | 9.00E-55 | 86.26 | 4 |
|  | one border found | supercontig_7 | 2320984 | 2321189 |  | 18866 | 18661 | 3.00E-100 | 97.57 | 7 |
|  | one border found | supercontig_9 | 2158403 | 2158546 |  | 154119 | 153977 | 3.00E-51 | 93.75 | 5 |
|  | one border found | supercontig_9 | 2158403 | 2158546 |  | 94731 | 94873 | 3.00E-51 | 93.75 | 5 |
|  | one border found | supercontig_11 | 934867 | 934979 |  | 150238 | 150126 | 3.00E-42 | 94.69 | 8 |
|  | one border found | supercontig_11 | 934867 | 934979 |  | 98612 | 98724 | 3.00E-42 | 94.69 | 8 |
|  | one border found | supercontig_11 | 2427206 | 2428804 |  | 59819 | 58220 | 0 | 94.07 | 8 |
|  | both borders found | supercontig_12 | 64846 | 64996 |  | 114723 | 114573 | 5.00E-65 | 96.03 | 4 |
|  | both borders found | supercontig_12 | 64846 | 64996 |  | 134127 | 134277 | 5.00E-65 | 96.03 | 4 |
|  | one border found | supercontig_13 | 469538 | 471349 |  | 124373 | 126178 | 0 | 94.12 | 9 |
|  | one border found | supercontig_15 | 866366 | 866657 |  | 155545 | 155837 | 8.00E-61 | 89.46 | 4 |
|  | one border found | supercontig_15 | 866366 | 866657 |  | 93305 | 93013 | 8.00E-61 | 89.46 | 4 |
| **Table S3. (Continued)** | | | | | | | | | | |
| **Type** | **Border Number** | **Scaffold** | **Insert Position** | |  | **Position in orgDNA** | | **E-value** | **Identity** | **Chromosome** |
|  |  |  | **Start** | **End** |  | **Start** | **End** |  |  |  |
|  | one border found | supercontig_16 | 2136292 | 2136559 |  | 112529 | 112799 | 1.00E-71 | 90.41 | 3 |
|  | one border found | supercontig_16 | 2136292 | 2136559 |  | 136321 | 136051 | 1.00E-71 | 90.41 | 3 |
|  | one border found | supercontig_17 | 2509534 | 2509642 |  | 102312 | 102420 | 2.00E-30 | 90.83 | 9 |
|  | one border found | supercontig_17 | 2509534 | 2509642 |  | 146538 | 146430 | 2.00E-30 | 90.83 | 9 |
|  | one border found | supercontig_18 | 386609 | 386788 |  | 111824 | 111645 | 9.00E-73 | 94.44 | 5 |
|  | one border found | supercontig_18 | 386609 | 386788 |  | 137026 | 137205 | 9.00E-73 | 94.44 | 5 |
|  | one border found | supercontig_22 | 1930257 | 1930370 |  | 131405 | 131292 | 2.00E-57 | 100 | 5 |
|  | one border found | supercontig_23 | 2635636 | 2635721 |  | 152003 | 152088 | 1.00E-28 | 94.19 | 7 |
|  | one border found | supercontig_23 | 2635636 | 2635721 |  | 96847 | 96762 | 1.00E-28 | 94.19 | 7 |
|  | both borders found | supercontig_25 | 1423654 | 1423756 |  | 67142 | 67245 | 3.00E-44 | 98.08 | 4 |
|  | both borders found | supercontig_26 | 1574754 | 1575148 |  | 153040 | 152647 | 0 | 98.99 | X/Y |
|  | both borders found | supercontig_26 | 1574754 | 1575148 |  | 95810 | 96203 | 0 | 98.99 | X/Y |
|  | one border found | supercontig_30 | 500302 | 500418 |  | 129493 | 129377 | 5.00E-40 | 93.16 | 2 |
|  | one border found | supercontig_30 | 879921 | 880111 |  | 69886 | 70077 | 5.00E-52 | 92.19 | 2 |
|  | one border found | supercontig_30 | 1101919 | 1102257 |  | 155991 | 155641 | 9.00E-131 | 95.73 | 2 |
|  | one border found | supercontig_30 | 1101919 | 1102257 |  | 92859 | 93209 | 9.00E-131 | 95.73 | 2 |
|  | one border found | supercontig_31 | 939865 | 940051 |  | 53789 | 53976 | 6.00E-49 | 88.83 | ND |
|  | one border found | supercontig_31 | 1279176 | 1279414 |  | 30010 | 29765 | 3.00E-66 | 94.72 | ND |
|  | both borders found | supercontig_33 | 1000440 | 1000759 |  | 61300 | 61618 | 4.00E-173 | 99.38 | 8 |
|  | one border found | supercontig_33 | 1713169 | 1713496 |  | 82579 | 82911 | 1.00E-151 | 95.8 | 8 |
|  | one border found | supercontig_35 | 695261 | 695444 |  | 3901 | 4087 | 2.00E-39 | 87.17 | 9 |
|  | one border found | supercontig_37 | 47515 | 47758 |  | 109742 | 109987 | 9.00E-41 | 85.37 | 6 |
|  | one border found | supercontig_37 | 47515 | 47758 |  | 139108 | 138863 | 9.00E-41 | 85.37 | 6 |
|  | one border found | supercontig_37 | 97733 | 97923 |  | 151226 | 151410 | 7.00E-57 | 95.29 | 6 |
|  | one border found | supercontig_37 | 97733 | 97923 |  | 97624 | 97440 | 7.00E-57 | 95.29 | 6 |
| **Table S3. (Continued)** | | | | | | | | | | |
| **Type** | **Border Number** | **Scaffold** | **Insert Position** | |  | **Position in orgDNA** | | **E-value** | **Identity** | **Chromosome** |
|  |  |  | **Start** | **End** |  | **Start** | **End** |  |  |  |
|  | one border found | supercontig_37 | 97931 | 98131 |  | 110316 | 110114 | 4.00E-80 | 95.59 | 6 |
|  | one border found | supercontig_37 | 97931 | 98131 |  | 138534 | 138736 | 4.00E-80 | 95.59 | 6 |
|  | one border found | supercontig_38 | 1655889 | 1655996 |  | 11965 | 11858 | 8.00E-54 | 100 | 2 |
|  | one border found | supercontig_40 | 859698 | 859801 |  | 104443 | 104546 | 2.00E-32 | 92.31 | 7 |
|  | one border found | supercontig_40 | 859698 | 859801 |  | 144407 | 144304 | 2.00E-32 | 92.31 | 7 |
|  | one border found | supercontig_40 | 859953 | 860053 |  | 104847 | 104946 | 2.00E-42 | 98.02 | 7 |
|  | one border found | supercontig_40 | 859953 | 860053 |  | 144003 | 143904 | 2.00E-42 | 98.02 | 7 |
|  | one border found | supercontig_41 | 1650414 | 1650610 |  | 110777 | 110974 | 4.00E-74 | 93.43 | 5 |
|  | one border found | supercontig_41 | 1650414 | 1650610 |  | 138073 | 137876 | 4.00E-74 | 93.43 | 5 |
|  | both borders found | supercontig_47 | 1192164 | 1192466 |  | 59440 | 59138 | 7.00E-125 | 93.73 | 2 |
|  | one border found | supercontig_49 | 1401580 | 1401782 |  | 34180 | 34382 | 7.00E-63 | 93.63 | X/Y |
|  | one border found | supercontig_55 | 475537 | 476022 |  | 152281 | 151796 | 3.00E-83 | 84.57 | 4 |
|  | one border found | supercontig_55 | 475537 | 476022 |  | 96569 | 97054 | 3.00E-83 | 84.57 | 4 |
|  | one border found | supercontig_56 | 833131 | 833616 |  | 41241 | 41725 | 5.00E-79 | 82.92 | 8 |
|  | one border found | supercontig_60 | 1471675 | 1471909 |  | 132350 | 132113 | 4.00E-58 | 87.82 | 6 |
|  | one border found | supercontig_63 | 645779 | 646077 |  | 153508 | 153207 | 3.00E-120 | 97.69 | ND |
|  | one border found | supercontig_63 | 645779 | 646077 |  | 95342 | 95643 | 3.00E-120 | 97.69 | ND |
|  | one border found | supercontig_63 | 1194839 | 1194940 |  | 57276 | 57378 | 2.00E-41 | 97.09 | ND |
|  | one border found | supercontig_65 | 1401473 | 1402134 |  | 18326 | 17664 | 0 | 99.4 | ND |
|  | both borders found | supercontig_78 | 215493 | 216019 |  | 2634 | 3159 | 0 | 98.29 | 7 |
|  | one border found | supercontig_88 | 196244 | 196308 |  | 127147 | 127211 | 1.00E-23 | 96.92 | ND |
|  | one border found | supercontig_103 | 763050 | 763355 |  | 101332 | 101019 | 1.00E-69 | 86.03 | 5 |
|  | one border found | supercontig_103 | 763050 | 763355 |  | 147518 | 147831 | 1.00E-69 | 86.03 | 5 |
|  | one border found | supercontig_106 | 484861 | 484991 |  | 152416 | 152546 | 7.00E-68 | 100 | 6 |
|  | one border found | supercontig_106 | 484861 | 484991 |  | 96434 | 96304 | 7.00E-68 | 100 | 6 |
| **Table S3. (Continued)** | | | | | | | | | | |
| **Type** | **Border Number** | **Scaffold** | **Insert Position** | |  | **Position in orgDNA** | | **E-value** | **Identity** | **Chromosome** |
|  |  |  | **Start** | **End** |  | **Start** | **End** |  |  |  |
|  | both borders found | supercontig_107 | 157083 | 157227 |  | 32784 | 32930 | 1.00E-53 | 94.56 | 9 |
|  | one border found | supercontig_109 | 310260 | 310462 |  | 11317 | 11520 | 3.00E-61 | 90.2 | ND |
|  | both borders found | supercontig_113 | 505664 | 505739 |  | 38113 | 38037 | 3.00E-26 | 96.1 | 2 |
|  | one border found | supercontig_119 | 154288 | 154354 |  | 152373 | 152307 | 5.00E-25 | 97.01 | 6 |
|  | one border found | supercontig_119 | 154288 | 154354 |  | 96477 | 96543 | 5.00E-25 | 97.01 | 6 |
|  | one border found | supercontig_125 | 882141 | 882344 |  | 109831 | 110036 | 1.00E-81 | 94.66 | 8 |
|  | one border found | supercontig_125 | 882141 | 882344 |  | 139019 | 138814 | 1.00E-81 | 94.66 | 8 |
|  | one border found | supercontig_131 | 667720 | 667990 |  | 12141 | 12409 | 2.00E-49 | 84.5 | 4 |
|  | one border found | supercontig_134 | 968918 | 969061 |  | 155080 | 154936 | 2.00E-62 | 97.95 | 9 |
|  | one border found | supercontig_134 | 968918 | 969061 |  | 93770 | 93914 | 2.00E-62 | 97.95 | 9 |
|  | one border found | supercontig_141 | 719179 | 719423 |  | 7595 | 7351 | 1.00E-47 | 84.9 | 5 |
|  | one border found | supercontig_143 | 231435 | 231646 |  | 110732 | 110946 | 9.00E-49 | 87.91 | ND |
|  | one border found | supercontig_143 | 231435 | 231646 |  | 138118 | 137904 | 9.00E-49 | 87.91 | ND |
|  | both borders found | supercontig_155 | 491419 | 491597 |  | 158759 | 158938 | 4.00E-69 | 95.03 | ND |
|  | both borders found | supercontig_155 | 491419 | 491597 |  | 90091 | 89912 | 4.00E-69 | 95.03 | ND |
|  | one border found | supercontig_185 | 355506 | 355627 |  | 73414 | 73293 | 6.00E-39 | 91.8 | ND |
|  | one border found | supercontig_185 | 448254 | 448470 |  | 75767 | 75979 | 5.00E-64 | 93.55 | ND |
|  | one border found | supercontig_195 | 53516 | 53999 |  | 108005 | 108487 | 1.00E-153 | 91.94 | ND |
|  | one border found | supercontig_195 | 53516 | 53999 |  | 140845 | 140363 | 1.00E-153 | 91.94 | ND |
|  | one border found | supercontig_209 | 166772 | 166930 |  | 33318 | 33475 | 2.00E-37 | 88.05 | 3 |
|  | one border found | supercontig_245 | 304142 | 304905 |  | 66523 | 65760 | 0 | 93.72 | 8 |
|  | one border found | supercontig_247 | 155226 | 155306 |  | 32313 | 32393 | 5.00E-29 | 95.06 | 6 |
|  | one border found | supercontig_367 | 46484 | 46600 |  | 36629 | 36745 | 1.00E-34 | 90.6 | ND |
|  | one border found | supercontig_389 | 26621 | 27310 |  | 105760 | 106450 | 0 | 89.15 | ND |
|  | one border found | supercontig_389 | 26621 | 27310 |  | 143090 | 142400 | 0 | 89.15 | ND |
| **Table S3. (Continued)** | | | | | | | | | | |
| **Type** | **Border Number** | **Scaffold** | **Insert Position** | |  | **Position in orgDNA** | | **E-value** | **Identity** | **Chromosome** |
|  |  |  | **Start** | **End** |  | **Start** | **End** |  |  |  |
|  | one border found | supercontig_469 | 25110 | 25314 |  | 51722 | 51927 | 6.00E-95 | 96.6 | ND |
|  | one border found | supercontig_519 | 32040 | 33169 |  | 126894 | 128024 | 0 | 97.35 | ND |
| fake* | one border found | contig_31780 | 1 | 2186 |  | 76623 | 78820 | 0 | 93.82 | ND |
|  | one border found | contig_33411 | 1 | 272 |  | 152834 | 153102 | 3.00E-98 | 93.8 | ND |
|  | one border found | contig_33411 | 1 | 272 |  | 96016 | 95748 | 3.00E-98 | 93.8 | ND |
|  | one border found | contig_33592 | 1 | 155 |  | 18175 | 18021 | 1.00E-48 | 90.32 | ND |
|  | one border found | contig_34595 | 1 | 63 |  | 62871 | 62933 | 2.00E-22 | 95.24 | ND |
|  | one border found | contig_37109 | 1 | 139 |  | 11929 | 12067 | 2.00E-75 | 100 | ND |
|  | one border found | contig_37937 | 1 | 161 |  | 158971 | 158811 | 3.00E-69 | 95.03 | ND |
|  | one border found | contig_37937 | 1 | 161 |  | 89879 | 90039 | 3.00E-69 | 95.03 | ND |
|  | one border found | contig_41959 | 1 | 1033 |  | 151241 | 150209 | 0 | 98.06 | ND |
|  | one border found | contig_41959 | 1 | 1033 |  | 97609 | 98641 | 0 | 98.06 | ND |
|  | one border found | contig_42072 | 1 | 962 |  | 82624 | 83585 | 0 | 100 | ND |
|  | one border found | contig_42427 | 1 | 136 |  | 63690 | 63825 | 5.00E-57 | 99.26 | ND |
|  | one border found | contig_42975 | 1 | 96 |  | 129846 | 129751 | 6.00E-45 | 97.92 | ND |
|  | one border found | contig_43403 | 1 | 189 |  | 108301 | 108489 | 4.00E-98 | 98.41 | ND |
|  | one border found | contig_43403 | 1 | 189 |  | 140549 | 140361 | 4.00E-98 | 98.41 | ND |
|  | one border found | contig_43443 | 1 | 296 |  | 109554 | 109257 | 1.00E-67 | 86.24 | ND |
|  | one border found | contig_43443 | 1 | 296 |  | 139296 | 139593 | 1.00E-67 | 86.24 | ND |
|  | one border found | contig_44028 | 1 | 91 |  | 154244 | 154154 | 1.00E-27 | 91.21 | ND |
|  | one border found | contig_44028 | 1 | 91 |  | 94606 | 94696 | 1.00E-27 | 91.21 | ND |
|  | one border found | contig_45536 | 1 | 222 |  | 27214 | 26993 | 2.00E-103 | 95.95 | ND |
|  | one border found | contig_45563 | 1 | 510 |  | 25413 | 24911 | 0 | 95.69 | ND |
|  | one border found | contig_47347 | 1 | 372 |  | 150548 | 150177 | 0 | 96.24 | ND |
|  | one border found | contig_47347 | 1 | 372 |  | 98302 | 98673 | 0 | 96.24 | ND |
| **Table S3. (Continued)** | | | | | | | | | | |
| **Type** | **Border Number** | **Scaffold** | **Insert Position** | |  | **Position in orgDNA** | | **E-value** | **Identity** | **Chromosome** |
|  |  |  | **Start** | **End** |  | **Start** | **End** |  |  |  |
|  | one border found | contig_48183 | 1 | 248 |  | 58583 | 58336 | 4.00E-115 | 96.39 | ND |
|  | one border found | supercontig_618 | 1 | 84 |  | 53594 | 53676 | 8.00E-23 | 94.12 | ND |
|  | one border found | supercontig_1200 | 1 | 148 |  | 26399 | 26252 | 2.00E-65 | 95.95 | ND |
|  | one border found | supercontig_1545 | 1 | 260 |  | 5519 | 5779 | 7.00E-102 | 93.1 | ND |
|  | one border found | supercontig_3885 | 1 | 1638 |  | 150528 | 152165 | 0 | 99.27 | ND |
|  | one border found | supercontig_3885 | 1 | 1638 |  | 98322 | 96685 | 0 | 99.27 | ND |
|  | one border found | supercontig_4671 | 1 | 104 |  | 133388 | 133491 | 3.00E-45 | 98.1 | ND |

Note: specific junction site positions in SunUp are highlighted in yellow; 'ND' indicates the corresponding chromosome data is unknown; (*): Scaffold positions of the nuclear genome scaffold start from the beginning.
