# Supplementary material for: Genomic variation between PRSV resistant transgenic SunUp and its progenitor cultivar Sunset
Source: BMC Genomics. 2020 Jun 12;21:398. doi: 10.1186/s12864-020-06804-7 (PMC7291442; doi:10.1186/s12864-020-06804-7)
Supplement: Supplementary file 4 — Additional file 4: Table S4. The genome positions of specific mitochondrion DNA integration sites in SunUp. [file 12864_2020_6804_MOESM4_ESM.docx]

Additional file 4: Table S4. The genome positions of specific mitochondrion DNA integration sites in SunUp

| **Type** | **Border Number** | **Scaffold** | **Insert Position** | |  | **Position in orgDNA** | | **E-value** | **Identity** | **Chromosome** |
| --- | --- | --- | --- | --- | --- | --- | --- | --- | --- | --- |
|  |  |  | **Start** | **End** |  | **start** | **end** |  |  |  |
| reliable | one border found | supercontig_0 | 308080 | 308217 |  | 7426 | 7570 | 2.00E-37 | 89.66 | 6 |
|  | one border found | supercontig_0 | 4223284 | 4223411 |  | 210318 | 210445 | 1.00E-26 | 87.5 | 6 |
|  | one border found | supercontig_1 | 731354 | 731960 |  | 163357 | 162751 | 0 | 95.39 | 2 |
|  | one border found | supercontig_1 | 2157338 | 2157501 |  | 111628 | 111465 | 1.00E-66 | 97.56 | 2 |
|  | one border found | supercontig_1 | 2158018 | 2158145 |  | 409354 | 409481 | 4.00E-48 | 94.53 | 2 |
|  | one border found | supercontig_2 | 1167088 | 1167532 |  | 239793 | 239357 | 3.00E-150 | 90.34 | 2 |
|  | one border found | supercontig_2 | 1167651 | 1168987 |  | 238722 | 237386 | 0 | 92.46 | 2 |
|  | both borders found | supercontig_2 | 2224101 | 2224227 |  | 423817 | 423943 | 3.00E-33 | 89.76 | 2 |
|  | one border found | supercontig_4 | 3420393 | 3420709 |  | 339230 | 338908 | 7.00E-86 | 89.47 | 9 |
|  | one border found | supercontig_5 | 1315770 | 1316121 |  | 211604 | 211955 | 6.00E-120 | 90.62 | 8 |
|  | one border found | supercontig_5 | 1539378 | 1539605 |  | 237215 | 236995 | 3.00E-78 | 91.23 | 8 |
|  | one border found | supercontig_8 | 855604 | 855736 |  | 390912 | 391044 | 7.00E-42 | 91.73 | 3 |
|  | both borders found | supercontig_8 | 1071513 | 1071660 |  | 63691 | 63844 | 1.00E-43 | 89.74 | 3 |
|  | one border found | supercontig_9 | 2158403 | 2158546 |  | 374278 | 374420 | 2.00E-48 | 93.06 | 5 |
|  | one border found | supercontig_10 | 2223924 | 2224051 |  | 137459 | 137586 | 3.00E-41 | 92.19 | 5 |
|  | one border found | supercontig_11 | 934867 | 934979 |  | 378142 | 378254 | 8.00E-42 | 94.69 | 8 |
|  | one border found | supercontig_11 | 2415566 | 2416010 |  | 83982 | 84426 | 5.00E-37 | 80.58 | 8 |
|  | one border found | supercontig_11 | 2571541 | 2572130 |  | 424849 | 424260 | 0 | 97.63 | 8 |
|  | one border found | supercontig_11 | 2572200 | 2572699 |  | 424084 | 423584 | 0 | 99.2 | 8 |
|  | one border found | supercontig_15 | 866366 | 866657 |  | 372873 | 372581 | 8.00E-54 | 87.41 | 4 |
|  | one border found | supercontig_17 | 2509534 | 2509642 |  | 238921 | 238813 | 2.00E-27 | 89.91 | 9 |
|  | both borders found | supercontig_21 | 1630016 | 1630122 |  | 344467 | 344361 | 3.00E-38 | 94.39 | X/Y |
|  | one border found | supercontig_23 | 2635636 | 2635721 |  | 376377 | 376292 | 4.00E-28 | 94.19 | 7 |
|  | both borders found | supercontig_26 | 1574754 | 1575131 |  | 375357 | 375734 | 3.00E-167 | 97.11 | X/Y |
| **Table S4. (Continued)** | | | | | | | | | | |
| **Type** | **Border Number** | **Scaffold** | **Insert Position** | |  | **Position in orgDNA** | | **E-value** | **Identity** | **Chromosome** |
|  |  |  | **Start** | **End** |  | **start** | **end** |  |  |  |
|  | one border found | supercontig_28 | 1016455 | 1016807 |  | 57020 | 56668 | 0 | 98.3 | ND |
|  | one border found | supercontig_30 | 735102 | 735577 |  | 134057 | 134533 | 2.00E-171 | 91.63 | 2 |
|  | one border found | supercontig_33 | 738275 | 738765 |  | 22724 | 23205 | 1.00E-169 | 91.45 | 8 |
|  | both borders found | supercontig_34 | 1410004 | 1410111 |  | 180067 | 180174 | 2.00E-44 | 98.17 | 5 |
|  | one border found | supercontig_35 | 448691 | 448837 |  | 202313 | 202162 | 5.00E-39 | 88.82 | 9 |
|  | one border found | supercontig_36 | 157916 | 158166 |  | 162004 | 161756 | 4.00E-107 | 95.24 | X/Y |
|  | one border found | supercontig_37 | 483553 | 484338 |  | 145507 | 146308 | 0 | 86.43 | 6 |
|  | one border found | supercontig_38 | 1173395 | 1177548 |  | 452665 | 448484 | 0 | 97.73 | 2 |
|  | one border found | supercontig_38 | 1177649 | 1178607 |  | 453904 | 452945 | 0 | 94.9 | 2 |
|  | one border found | supercontig_40 | 859677 | 859801 |  | 236957 | 236844 | 4.00E-24 | 85.6 | 7 |
|  | one border found | supercontig_40 | 859953 | 860053 |  | 236530 | 236431 | 3.00E-37 | 96.04 | 7 |
|  | one border found | supercontig_40 | 1295820 | 1296030 |  | 463187 | 463397 | 5.00E-110 | 99.05 | 7 |
|  | one border found | supercontig_46 | 1165752 | 1165815 |  | 94435 | 94372 | 2.00E-22 | 96.88 | 7 |
|  | one border found | supercontig_46 | 1165811 | 1165898 |  | 32124 | 32211 | 1.00E-36 | 97.73 | 7 |
|  | one border found | supercontig_49 | 885454 | 885524 |  | 199194 | 199264 | 5.00E-29 | 98.59 | X/Y |
|  | one border found | supercontig_50 | 842216 | 842533 |  | 319172 | 318856 | 5.00E-128 | 95.28 | 3 |
|  | one border found | supercontig_55 | 475537 | 476022 |  | 376099 | 376584 | 2.00E-68 | 83.33 | 4 |
|  | one border found | supercontig_56 | 716004 | 716704 |  | 237324 | 236627 | 0 | 90.08 | 8 |
|  | one border found | supercontig_60 | 1470886 | 1471573 |  | 27754 | 28443 | 6.00E-152 | 85.8 | 6 |
|  | one border found | supercontig_61 | 305951 | 306285 |  | 23948 | 23614 | 1.00E-131 | 92.84 | 7 |
|  | one border found | supercontig_61 | 1353833 | 1354033 |  | 232638 | 232840 | 4.00E-36 | 85.71 | 7 |
|  | both borders found | supercontig_63 | 498377 | 498499 |  | 189601 | 189720 | 8.00E-34 | 92.68 | ND |
|  | one border found | supercontig_63 | 645779 | 646077 |  | 374889 | 375190 | 1.00E-112 | 96.7 | ND |
|  | one border found | supercontig_68 | 376666 | 378132 |  | 125211 | 126687 | 0 | 94.04 | ND |
|  | one border found | supercontig_68 | 745444 | 748316 |  | 253065 | 250189 | 0 | 93.58 | ND |
| **Table S4. (Continued)** | | | | | | | | | | |
| **Type** | **Border Number** | **Scaffold** | **Insert Position** | |  | **Position in orgDNA** | | **E-value** | **Identity** | **Chromosome** |
|  |  |  | **Start** | **End** |  | **start** | **end** |  |  |  |
|  | one border found | supercontig_68 | 749407 | 750166 |  | 249262 | 248503 | 0 | 93.16 | ND |
|  | both borders found | supercontig_73 | 206209 | 206343 |  | 223684 | 223822 | 2.00E-22 | 87.05 | 6 |
|  | both borders found | supercontig_73 | 206209 | 206343 |  | 470769 | 470907 | 2.00E-22 | 87.05 | 6 |
|  | one border found | supercontig_73 | 1157451 | 1157783 |  | 140032 | 139699 | 1.00E-134 | 95.21 | 6 |
|  | one border found | supercontig_75 | 1271615 | 1271805 |  | 365624 | 365424 | 1.00E-57 | 89.6 | 7 |
|  | one border found | supercontig_80 | 631770 | 631884 |  | 70785 | 70667 | 3.00E-39 | 94.96 | 4 |
|  | one border found | supercontig_82 | 1190704 | 1190886 |  | 174751 | 174934 | 5.00E-32 | 85.33 | ND |
|  | one border found | supercontig_83 | 667528 | 667666 |  | 74832 | 74971 | 5.00E-56 | 95.71 | 3 |
|  | one border found | supercontig_84 | 925135 | 925219 |  | 78371 | 78454 | 5.00E-28 | 95.29 | 3 |
|  | one border found | supercontig_87 | 870750 | 871263 |  | 387790 | 387277 | 0 | 96.11 | 3 |
|  | one border found | supercontig_88 | 665858 | 665934 |  | 390379 | 390303 | 8.00E-21 | 92.21 | ND |
|  | one border found | supercontig_96 | 321280 | 321624 |  | 153467 | 153122 | 0 | 98.55 | ND |
|  | one border found | supercontig_100 | 764126 | 764414 |  | 113369 | 113081 | 5.00E-149 | 100 | ND |
|  | one border found | supercontig_100 | 764414 | 764520 |  | 338260 | 338154 | 5.00E-53 | 100 | ND |
|  | one border found | supercontig_102 | 756358 | 756703 |  | 223778 | 223433 | 6.00E-179 | 97.98 | ND |
|  | one border found | supercontig_102 | 756358 | 756703 |  | 470863 | 470518 | 6.00E-179 | 97.98 | ND |
|  | one border found | supercontig_103 | 763221 | 763355 |  | 380669 | 380536 | 1.00E-31 | 88.89 | 5 |
|  | one border found | supercontig_104 | 442724 | 442911 |  | 384608 | 384421 | 2.00E-94 | 98.4 | 8 |
|  | both borders found | supercontig_106 | 702697 | 702837 |  | 228529 | 228669 | 3.00E-42 | 90.78 | 6 |
|  | both borders found | supercontig_106 | 702697 | 702837 |  | 475614 | 475754 | 3.00E-42 | 90.78 | 6 |
|  | one border found | supercontig_118 | 64601 | 64871 |  | 224338 | 224610 | 3.00E-44 | 85.35 | 2 |
|  | one border found | supercontig_118 | 64601 | 64871 |  | 471423 | 471695 | 3.00E-44 | 85.35 | 2 |
|  | one border found | supercontig_119 | 154288 | 154354 |  | 376007 | 376073 | 3.00E-22 | 95.52 | 6 |
|  | one border found | supercontig_120 | 380943 | 381385 |  | 174100 | 174554 | 0 | 92.53 | 8 |
|  | both borders found | supercontig_129 | 250172 | 250259 |  | 118172 | 118085 | 4.00E-25 | 92.05 | 4 |
| **Table S4. (Continued)** | | | | | | | | | | |
| **Type** | **Border Number** | **Scaffold** | **Insert Position** | |  | **Position in orgDNA** | | **E-value** | **Identity** | **Chromosome** |
|  |  |  | **Start** | **End** |  | **start** | **end** |  |  |  |
|  | one border found | supercontig_129 | 555260 | 555332 |  | 224317 | 224389 | 2.00E-23 | 94.52 | 4 |
|  | one border found | supercontig_129 | 555260 | 555332 |  | 471402 | 471474 | 2.00E-23 | 94.52 | 4 |
|  | one border found | supercontig_131 | 666861 | 666980 |  | 138578 | 138459 | 1.00E-46 | 95 | 4 |
|  | one border found | supercontig_134 | 265423 | 265724 |  | 439283 | 439585 | 1.00E-112 | 92.41 | 9 |
|  | one border found | supercontig_134 | 266094 | 266191 |  | 439593 | 439690 | 1.00E-35 | 94.9 | 9 |
|  | one border found | supercontig_134 | 968918 | 969061 |  | 373317 | 373461 | 1.00E-59 | 97.26 | 9 |
|  | one border found | supercontig_136 | 869791 | 869852 |  | 161810 | 161749 | 4.00E-26 | 100 | 3 |
|  | one border found | supercontig_142 | 679552 | 679750 |  | 239793 | 239602 | 5.00E-52 | 87.94 | X/Y |
|  | one border found | supercontig_155 | 312138 | 312247 |  | 131135 | 131244 | 2.00E-30 | 95.45 | ND |
|  | both borders found | supercontig_155 | 491422 | 491597 |  | 369655 | 369479 | 7.00E-67 | 94.94 | ND |
|  | both borders found | supercontig_166 | 224074 | 224233 |  | 240621 | 240781 | 1.00E-54 | 92.55 | 9 |
|  | both borders found | supercontig_180 | 239430 | 239658 |  | 269883 | 269655 | 1.00E-73 | 90.39 | 4 |
|  | one border found | supercontig_182 | 129948 | 130232 |  | 416355 | 416639 | 8.00E-133 | 97.89 | 8 |
|  | one border found | supercontig_183 | 193632 | 193739 |  | 283142 | 283035 | 3.00E-30 | 90.74 | 2 |
|  | one border found | supercontig_193 | 326412 | 326682 |  | 30835 | 30564 | 2.00E-109 | 94.51 | 5 |
|  | one border found | supercontig_198 | 160791 | 160914 |  | 132467 | 132344 | 8.00E-64 | 100 | 7 |
|  | one border found | supercontig_232 | 120901 | 121042 |  | 178166 | 178309 | 3.00E-37 | 90.28 | 6 |
|  | one border found | supercontig_248 | 104319 | 104508 |  | 381895 | 382084 | 2.00E-79 | 94.74 | 6 |
|  | one border found | supercontig_248 | 104601 | 104713 |  | 382080 | 382193 | 4.00E-41 | 94.74 | 6 |
|  | one border found | supercontig_296 | 118503 | 118842 |  | 337962 | 338302 | 3.00E-145 | 95.6 | 4 |
|  | one border found | supercontig_328 | 109885 | 109950 |  | 226132 | 226066 | 2.00E-25 | 98.51 | ND |
|  | one border found | supercontig_328 | 109885 | 109950 |  | 473217 | 473151 | 2.00E-25 | 98.51 | ND |
|  | one border found | supercontig_745 | 27746 | 27909 |  | 300143 | 300306 | 7.00E-72 | 95.73 | ND |
| fake* | one border found | contig_33673 | 1 | 532 |  | 201626 | 201095 | 0 | 98.87 | ND |
|  | one border found | contig_34754 | 1 | 103 |  | 287362 | 287260 | 8.00E-51 | 99.03 | ND |
| **Table S4. (Continued)** | | | | | | | | | | |
| **Type** | **Border Number** | **Scaffold** | **Insert Position** | |  | **Position in orgDNA** | | **E-value** | **Identity** | **Chromosome** |
|  |  |  | **Start** | **End** |  | **start** | **end** |  |  |  |
|  | one border found | contig_34983 | 1 | 95 |  | 272166 | 272261 | 2.00E-39 | 96.88 | ND |
|  | one border found | contig_35418 | 1 | 668 |  | 296764 | 297431 | 0 | 98.8 | ND |
|  | one border found | contig_36228 | 1 | 240 |  | 64338 | 64099 | 4.00E-71 | 89.63 | ND |
|  | one border found | contig_37401 | 1 | 217 |  | 58061 | 57848 | 1.00E-61 | 95.87 | ND |
|  | one border found | contig_37937 | 1 | 161 |  | 369446 | 369606 | 5.00E-64 | 93.79 | ND |
|  | one border found | contig_38228 | 1 | 1073 |  | 83527 | 82454 | 0 | 98.51 | ND |
|  | one border found | contig_39860 | 1 | 234 |  | 434770 | 435003 | 8.00E-132 | 100 | ND |
|  | one border found | contig_40869 | 1 | 235 |  | 273091 | 272857 | 6.00E-94 | 93.19 | ND |
|  | one border found | contig_41391 | 1 | 97 |  | 152013 | 151917 | 7.00E-38 | 94.85 | ND |
|  | one border found | contig_41959 | 1 | 1033 |  | 377139 | 378171 | 0 | 97.58 | ND |
|  | one border found | contig_44028 | 1 | 91 |  | 374153 | 374243 | 2.00E-22 | 89.01 | ND |
|  | one border found | contig_44758 | 1 | 87 |  | 162437 | 162351 | 3.00E-39 | 97.7 | ND |
|  | one border found | contig_47347 | 1 | 372 |  | 377832 | 378203 | 2.00E-178 | 95.97 | ND |
|  | one border found | supercontig_1446 | 1 | 414 |  | 142650 | 143063 | 0 | 98.55 | ND |
|  | one border found | supercontig_1943 | 1 | 759 |  | 298084 | 298842 | 0 | 98.55 | ND |
|  | one border found | supercontig_3469 | 1 | 1066 |  | 352094 | 353157 | 0 | 97.84 | ND |
|  | one border found | supercontig_3885 | 1 | 1638 |  | 377852 | 376215 | 0 | 98.66 | ND |

Note: specific junction site positions in SunUp are highlighted in yellow; 'ND' indicates the corresponding chromosome data is unknown; (*): Scaffold positions of the nuclear genome scaffold start from the beginning.
