# Supplementary material for: Genomic variation between PRSV resistant transgenic SunUp and its progenitor cultivar Sunset
Source: BMC Genomics. 2020 Jun 12;21:398. doi: 10.1186/s12864-020-06804-7 (PMC7291442; doi:10.1186/s12864-020-06804-7)
Supplement: Supplementary file 5 — Additional file 5: Figure S1. Chromosomal and genome-wide distribution of the frequency of A. SNP/InDels B. SNPs and C. InDels per 1 kb in the Sunset genome compared to the SunUp reference genome. [file 12864_2020_6804_MOESM5_ESM.docx]

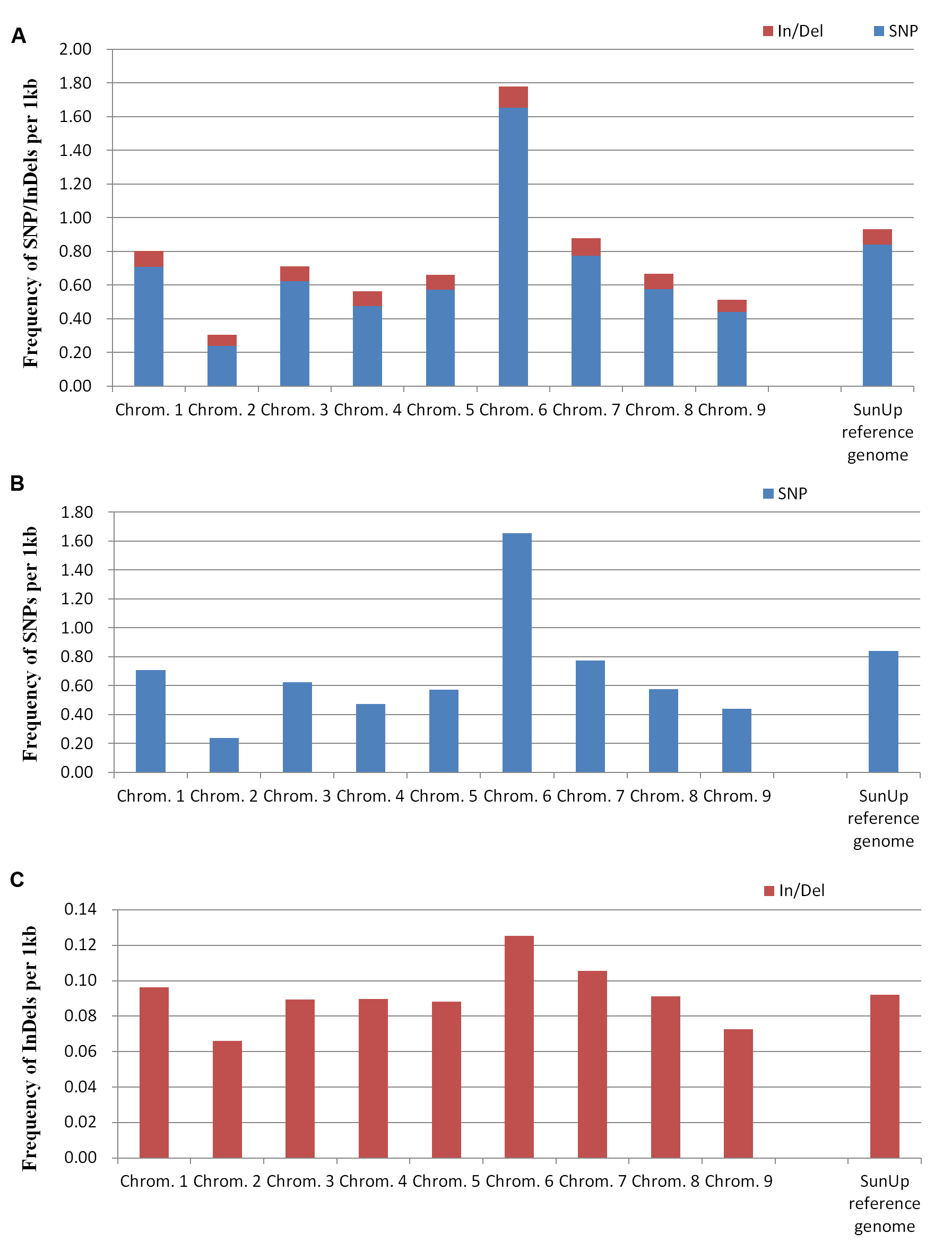


Additional file 5: Fig. S1 Chromosomal and genome-wide distribution of the frequency of A. SNP/InDels B. SNPs and C. InDels per 1kb in the Sunset genome compared to the SunUp reference genome
