# Supplementary material for: Genomic variation between PRSV resistant transgenic SunUp and its progenitor cultivar Sunset
Source: BMC Genomics. 2020 Jun 12;21:398. doi: 10.1186/s12864-020-06804-7 (PMC7291442; doi:10.1186/s12864-020-06804-7)
Supplement: Supplementary file 6 — Additional file 6: Figure S2. Transitions and transversions of homo and hetero SNPs identified in the Sunset genome compared to the SunUp reference genome. A. Frequency of different nucleotide substitution types in homo and hetero SNPs. B. Numbers and percentages of transitions (Ts) and transversions (Tv), and the Ts/Tv ratio in homo/hetero SNPs and total SNPs. [file 12864_2020_6804_MOESM6_ESM.docx]

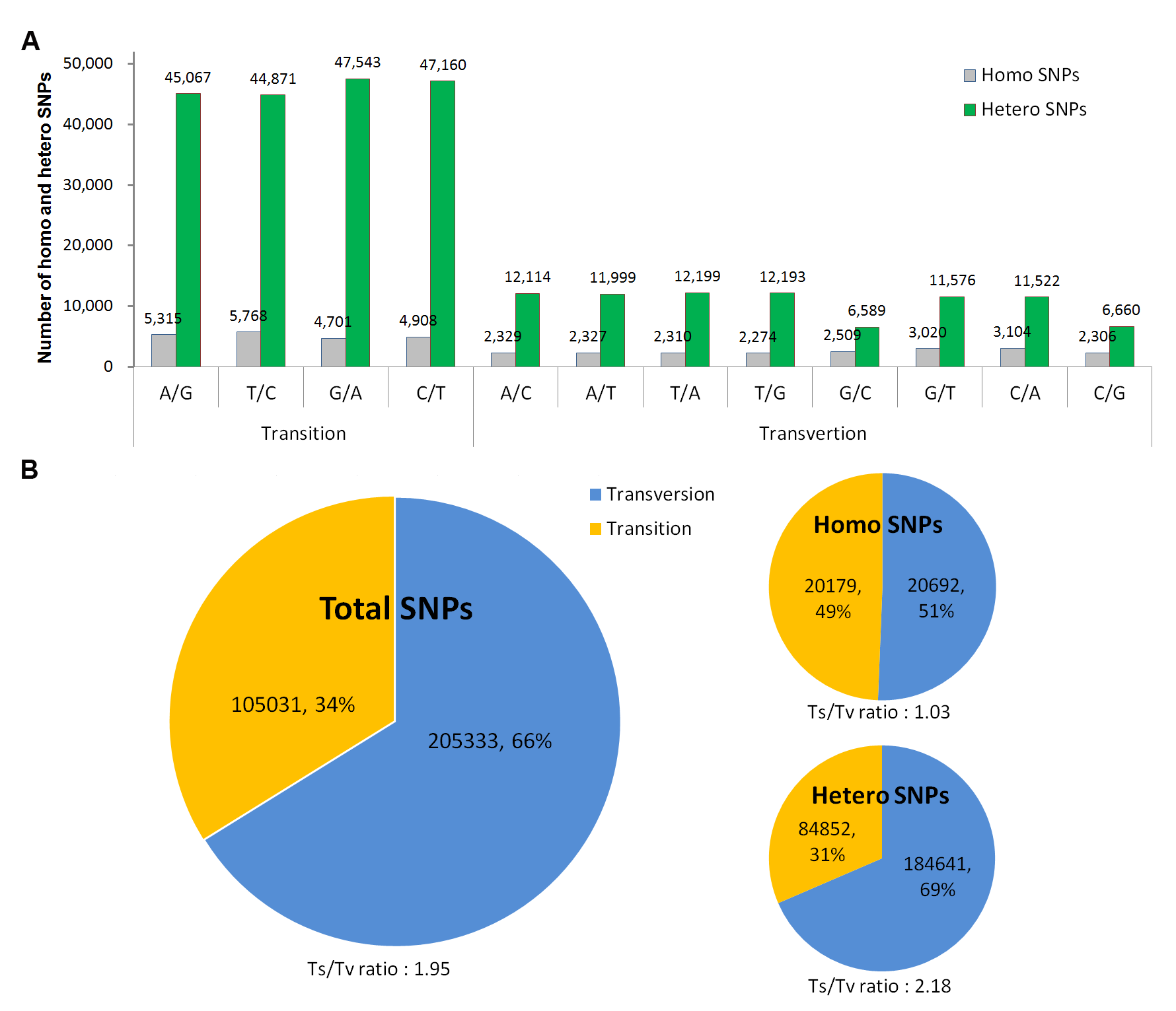


Additional file 6: Fig. S2 Transitions and transversions of homo and hetero SNPs identified in the Sunset genome compared to the SunUp reference genome. A. Frequency of different nucleotide substitution types in homo and hetero SNPs. B. Numbers and percentages of transitions (Ts) and transversions (Tv), and the Ts/Tv ratio in homo/hetero SNPs and total SNPs.
