# Supplementary material for: Genomic variation between PRSV resistant transgenic SunUp and its progenitor cultivar Sunset
Source: BMC Genomics. 2020 Jun 12;21:398. doi: 10.1186/s12864-020-06804-7 (PMC7291442; doi:10.1186/s12864-020-06804-7)
Supplement: Supplementary file 7 — Additional file 7: Figure S3. AgriGO results of putative high mutation genes based on SEA analysis. Significance level of enrichment is displayed using a color scale. White indicates no significant enrichment; color transitions from yellow to red indicate an increase in the strength of significance. Ratios at the bottom of each GO box represent the number of genes in the input list matching that GO term versus the number of total genes in the input list, total genes in the background genome set matching that GO term versus total genes in the background set. The adjusted p-value for each enriched GO term is indicated in parentheses at the top of each colored box. [file 12864_2020_6804_MOESM7_ESM.docx]

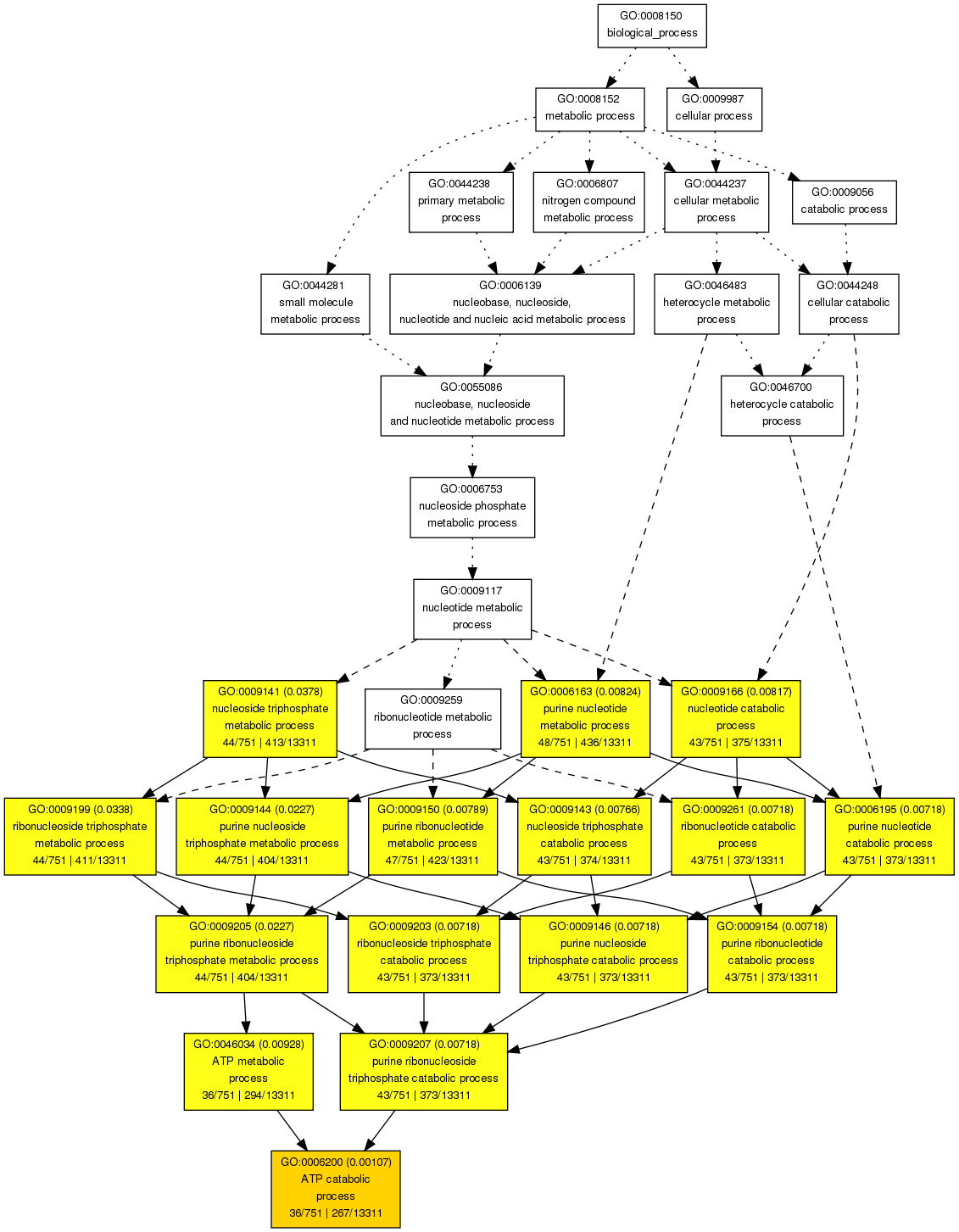


Additional file 7: Fig. S3 AgriGO results of putative high mutation genes based on SEA analysis. Significance level of enrichment is displayed using a color scale. White indicates no significant enrichment; color transitions from yellow to red indicate an increase in the strength of significance. Ratios at the bottom of each GO box represent the number of genes in the input list matching that GO term versus the number of total genes in the input list, total genes in the background genome set matching that GO term versus total genes in the background set. The adjusted *p*-value for each enriched GO term is indicated in parentheses at the top of each colored box.
